# Comparing the evidential strength for psychotropic drugs: a Bayesian meta-analysis

**DOI:** 10.1017/S0033291721003950

**Published:** 2021-12

**Authors:** Merle-Marie Pittelkow, Ymkje Anna de Vries, Rei Monden, Jojanneke A. Bastiaansen, Don van Ravenzwaaij

**Affiliations:** 1 Department Psychometrics and Statistics, University of Groningen, Groningen, the Netherlands; 2 Department of Developmental Psychology, University of Groningen, Groningen, the Netherlands; 3 Interdisciplinary Center Psychopathology and Emotion Regulation, Department of Psychiatry, University Medical Center Groningen, Groningen, the Netherlands; 4 Department of Biomedical Statistics, Graduate School of Medicine, Osaka University, Suita, Osaka, Japan; 5 Department of Education and Research, Friesland Mental Health Care Services, Leeuwarden, the Netherlands

**Keywords:** Bayesian meta-analysis, drug endorsement, evidential strength, psychotropic drugs

## Abstract

Approval and prescription of psychotropic drugs should be informed by the strength of evidence for efficacy. Using a Bayesian framework, we examined (1) whether psychotropic drugs are supported by substantial evidence (at the time of approval by the Food and Drug Administration), and (2) whether there are systematic differences across drug groups. Data from short-term, placebo-controlled phase II/III clinical trials for 15 antipsychotics, 16 antidepressants for depression, nine antidepressants for anxiety, and 20 drugs for attention deficit hyperactivity disorder (ADHD) were extracted from FDA reviews. Bayesian model-averaged meta-analysis was performed and strength of evidence was quantified (i.e. *BF*_BMA_). Strength of evidence and trialling varied between drugs. Median evidential strength was extreme for ADHD medication (*BF*_BMA_ = 1820.4), moderate for antipsychotics (*BF*_BMA_ = 365.4), and considerably lower and more frequently classified as weak or moderate for antidepressants for depression (*BF*_BMA_ = 94.2) and anxiety (*BF*_BMA_ = 49.8). Varying median effect sizes (*ES*_schizophrenia_ = 0.45, *ES*_depression_ = 0.30, *ES*_anxiety_ = 0.37, *ES*_ADHD_ = 0.72), sample sizes (*N*_schizophrenia_ = 324, *N*_depression_ = 218, *N*_anxiety_ = 254, *N*_ADHD_ = 189.5), and numbers of trials (*k*_schizophrenia_ = 3, *k*_depression_ = 5.5, *k*_anxiety_ = 3, *k*_ADHD_ = 2) might account for differences. Although most drugs were supported by strong evidence at the time of approval, some only had moderate or ambiguous evidence. These results show the need for more systematic quantification and classification of statistical evidence for psychotropic drugs. Evidential strength should be communicated transparently and clearly towards clinical decision makers.

## Background

Psychiatric disorders can be treated with various psychotropic drugs. With a wide variety of drugs available, choosing the most appropriate one can be difficult, highlighting the importance of good evidence. Clinicians must be able to trust that there is strong evidence that the drug is effective. In the USA, drugs must be approved by the Food and Drug Administration (FDA) before they can be marketed. Although many aspects of a drug's profile must be considered in the approval process, the statistical evaluation of efficacy plays a central role. The FDA states that substantial evidence for efficacy is provided by ‘at least two adequate and well-controlled studies, each convincing on their own’ (U.S. Food and Drug Administration, 1998, p. 3). Occasionally, efficacy can also be established based on ‘data from one adequate, well-controlled clinical investigation’ (U.S. Food and Drug Administration, 1998, p. 3) or existing efficacy studies of closely related drugs, for example for modified-release variants of previously approved drugs (U.S. Food and Drug Administration, 1997, 1998; Wang et al., 2019).

In some cases, the current statistical evaluation process may lead to suboptimal decisions. The assumption that at least two independent randomised controlled trials (RCTs), or even fewer, provide substantial evidence of drug efficacy has been questioned (Monden et al., 2018). The FDA decision process does not systematically combine the information from positive (i.e. *p* < 0.05, rejecting the null hypothesis of no treatment effect) and negative (i.e. *p* > 0.05, not rejecting the null hypothesis) trials. As such, crucial information like the number of trials conducted before obtaining two positive trials is ignored (van Ravenzwaaij & Ioannidis, 2017, 2019). Instead, the Bayes factor (*BF*) has been suggested as a measure to quantify evidence holistically (Goodman, 1999; Monden et al., 2016, 2018). In contrast to *p* values, *BF*s quantify evidence in favour of both the null hypothesis (i.e. no treatment effect) and the alternative hypothesis (i.e. a treatment effect) by comparing the relative likelihood of the observed data under either hypothesis (Gronau, Ly, & Wagenmakers, 2019; Jeffreys, 1961; Rouder, Speckman, Sun, Morey, & Iverson, 2009; van Ravenzwaaij & Ioannidis, 2019). For instance, a *BF*_10_ (where the subscript indicates that the *BF* quantifies the likelihood of the alternative hypothesis relative to the null hypothesis) of 30 indicates the observed data are 30 times more likely to have occurred under the alternative hypothesis than under the null hypothesis (this is considered strong evidence for the alternative hypothesis; Jeffreys, 1961). Alternatively, a *BF*_10_ of 0.2 (or 1/5) indicates the observed data are five times more likely to have occurred under the null hypothesis than under the alternative hypothesis. Finally, a *BF* around 1 indicates equipoise (i.e. the data are about equally likely to have occurred under either hypothesis).

*BF*s may not only aid drug approval, but also drug prescription by clinicians. Besides effect sizes, which indicate the *magnitude* of the effect (Sullivan & Feinn, 2012), strength of evidence as quantified through *BF*s indicates how likely an effect (of any positive size) is to exist. Ideally, effect sizes should be clinically meaningful and the strength of evidence sufficiently large that the drug can be considered effective with relative certainty. There is a great body of literature regarding effect sizes of psychotropic drugs (Cipriani et al., 2018; Cortese et al., 2018; Huhn et al., 2019; Leucht, Helfer, Gartlehner, & Davis, 2015). However, little is known about the evidential strength of these drugs, which can differ despite homogeneous effect sizes. For example, in a previous study adopting a Bayesian framework, sertraline, fluoxetine, and desvenlafaxine had similar estimated effect sizes, but strength of evidence differed by a factor of two (Monden et al., 2018). Especially in situations such as these, *BF*s can offer an important additional source of information.

Evidential strength might differ between psychotropic drug groups as well as within. Previous research has shown that there are clear differences among psychotropic drug groups in terms of effect size (Leucht et al., 2015; Turner, Knoepflmacher, & Shapley, 2012). There are also indications that trial programmes differ between drug groups: for instance, drug approvals of antidepressants for anxiety disorders were generally supported by fewer trials than approvals of antidepressants for depression (Roest et al., 2015; Turner, Matthews, Linardatos, Tell, & Rosenthal, 2008). Although, to the best of our knowledge, there is no formal policy towards different standards for drug approval, these differences may lead to differences in the typical strength of evidence for different drug groups. However, little is known about the extent to which such factors influence the typical strength of evidence for different drug groups.

### The present study

The goal of this study is to examine whether there are systematic differences in the strength of evidence for efficacy at the time of approval between different groups of psychotropic drugs. We consider four major classes: antidepressants approved for depression, antidepressants approved for anxiety disorders, antipsychotics for schizophrenia, and attention deficit hyperactivity disorder (ADHD) medication. We examine whether the current evaluation process generally leads to psychotropic drugs supported by substantial evidence (at the time of approval). To determine whether there are systematic differences across drug groups in terms of strength of evidence, we compare them across the disorder groups and investigate whether trial programme characteristics (e.g. effect sizes and sample sizes) are related to the strength of evidence per drug within each drug group.

## Method

This study involved publicly available trial-level data. No ethical approval was needed.

### Protocol and registration

Study information, details regarding prior knowledge of the data, and the analysis plan were preregistered at OSF before data analysis but after knowledge of the data. Deviations from the preregistration are reported in the online Supplementary material.

### Data sources

Data sources were not identified by a systematic search. Instead, we obtained data for psychotropic drugs approved by the FDA. We used data extracted for previous meta-analyses, supplemented by data extracted by ourselves. Data on antidepressants for depression were obtained from Turner et al. (2008) and de Vries et al. (2018), on antidepressants for anxiety disorders from de Vries, de Jonge, Van Heuvel, Turner, and Roest (2016) and Roest et al. (2015), and on antipsychotics for schizophrenia from Turner et al. (2012). We extracted additional data on medications for ADHD, and antipsychotics for schizophrenia approved after publication of Turner et al. (2012) from FDA reviews. No additional data extraction was necessary for depression or anxiety disorders, as no new antidepressants were approved for these indications after previous publications. We followed data extraction procedures originally proposed by Turner et al. (2008) described in detail elsewhere (Turner et al., 2012). In short, for each drug we retrieved the corresponding FDA reviews from the FDA's website. Within the Drug Approval package, data relevant to the FDA's determination of drug efficacy were examined. Clinical phase II/III trials pivotal in the endorsement decision of the drug were eligible for inclusion, regardless of their outcome. Efficacy data were extracted preferably from the statistical review, and from the medical review or team leader memos, if necessary. In total, we included data for 15 antipsychotics (*Nr*_trials_ = 43, *n*_treatment_ = 9937, *n*_control_ = 4303), 16 antidepressants approved for depression (*Nr*_trials_ = 105, *n*_treatment_ = 14 042, *n*_control_ = 9917), nine antidepressants approved for anxiety (*Nr*_trials_ = 59, *n*_treatment_ = 8745, *n*_control_ = 6618), and 20 drugs approved for ADHD (*Nr*_trials_ = 46, *n*_treatment_ = 5705, *n*_control_ = 3508).^†^^1^ For anxiety, we focused on generalised anxiety disorder (GAD), obsessive compulsive disorder (OCD), panic disorder (PD), post-traumatic stress disorder (PTSD), and social anxiety disorder (SAD). Some drugs were approved for multiple anxiety disorders. Consequently, we included 21 endorsement decisions for anxiety, resulting in a total of 72 drug-disorder combinations.

We included data for all available short-term, placebo-controlled, parallel-group, and cross-over phase II/III clinical trials. We excluded studies concerned with relapse or discontinuation of the medication, long-term extension trials, and studies without a placebo control group, as these do not qualify as ‘well-controlled’ trials (U.S. Food and Drug Administration, 2020). We excluded data on non-approved sub-therapeutic dosages (i.e. not effective dosages), as we were concerned with the evidence load regarding dosages associated with a therapeutic effect.

### Statistical analysis

Analysis was conducted in R (4.0.1), using the ‘BayesFactor’ (0.9.12–4.2) (Morey, Rouder, Jamil, & Morey, 2015) and ‘metaBMA’ (Heck, Gronau, & Wagenmakers, 2017) packages.

#### Individual *BF* and effect size calculation

We calculated *t* statistics using sample size and *p* values. For parallel-group trials, we used independent samples *t* tests and for cross-over trials, we used paired samples *t* tests (Higgins et al., 2019). We used two-sided tests, in concordance with the FDA policy. Following Monden et al. (2018), we calculated a *t* statistic for all dose levels in fixed-dose trials with multiple drug arms, whereas one *t* value was calculated for flexible-dose trials with a single drug arm. When precise *p* values were unavailable, *t* statistics were calculated based on other information (e.g. mean differences) or imputed (see online Supplementary material). To determine the strength of evidence that an effect exists, we calculated Jeffreys–Zellner–Siow *BF*s (Rouder et al., 2009; van Ravenzwaaij & Etz, 2020). For each comparison, *BF*s were calculated using *t* statistics and sample size of the drug and placebo groups. We used a default Cauchy prior with location parameter zero and scale parameter 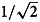 (Bayarri, Berger, Forte, & García-Donato, 2012; Consonni, Fouskakis, Liseo, & Ntzoufras, 2018). As the FDA follows two-sided tests with a check for direction, thus *de facto* performing a one-sided test, we truncated below zero, and calculated one-sided *BF*s (Senn, 2008).

To determine the size of an effect, we calculated the standardised mean difference (SMD). For parallel group trials, we calculated the corrected Hedges *g*. For cross-over trials, the uncorrected SMD was used.

#### Model-averaged Bayesian meta-analysis

We implemented Bayesian model-averaging (BMA; Gronau, Heck, Berkhout, Haaf, & Wagenmakers, 2021), as neither a fixed-effect model (assuming the same underlying ‘true’ effect-size) nor a random-effect model (being overly complex for meta-analysis of only a handful of trials) was believed to be necessarily best-suited for the present data. Instead, we weighted the results from both models according to their posterior probability, thus fully acknowledging the uncertainty with respect to the choice between a fixed or random-effect model (Gronau et al., 2017; Hinne, Gronau, van den Bergh, & Wagenmakers, 2020).

To conduct a Bayesian meta-analysis, prior distributions were assigned to the model parameters (Gronau et al., 2017). For the standardised effect size, we used a default, zero-centred Cauchy distribution with scale parameter equal to 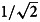 (Morey et al., 2015). For the one-sided hypothesis test, we used the same distribution with values below zero truncated. For the between-study heterogeneity parameter *τ* in random-effect models, we used an informed prior distribution based on an analysis of 14 886 meta-analyses from the Cochrane Database of Systematic Reviews (Turner, Davey, Clarke, Thompson, & Higgins, 2012, 2015), namely a log normal distribution with mean *μ* = *−* 2.12 and standard deviation s.d. = 1.532. We performed one Bayesian meta-analysis per endorsement decision. This yielded pooled estimates of both effect size and strength of evidence for the effects (i.e. efficacy of a certain drug for a specific mental disorder), in the form of model-averaged *BF*s (*BF*_BMA_). A total of 63 Bayesian meta-analyses were performed. For nine drug-disorder combinations supported by a single two-arm trial, we did not conduct a Bayesian meta-analysis, but used the individual *BF* and effect size instead.^2^ Hence, results are reported for 72 drug-disorder combinations.

The resulting *BF*_BMA_ were used to describe the proportion of well-supported endorsement decisions. We used different thresholds to quantify ‘substantial’ evidence. A *BF*_10_ between 1/3 and 3 is interpreted as ambiguous evidence, while a *BF*_10_ between 3 and 10 provides moderate, a *BF*_10_ between 10 and 30 strong, and a *BF*_10_ above 30 very strong evidence for the treatment effect (Jeffreys, 1961). Importantly, these thresholds are used for demonstrative purposes and we did not aim for just another hard threshold such as *p* < 0.05 (see Gelman, 2015).

#### Sensitivity analysis

To study the impact of the choices we made for the prior distributions on outcomes, we performed a sensitivity analysis by setting parameter estimates varied from 
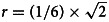
 to 
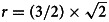
. Additionally, we inspected the differences between fixed-effect and random-effect models and how excluding imputed values or cross-over trials impacted the results. Details can be found in the online Supplementary material.

## Results

### Proportion of studies supported by substantial evidence

**Fig. 1.**
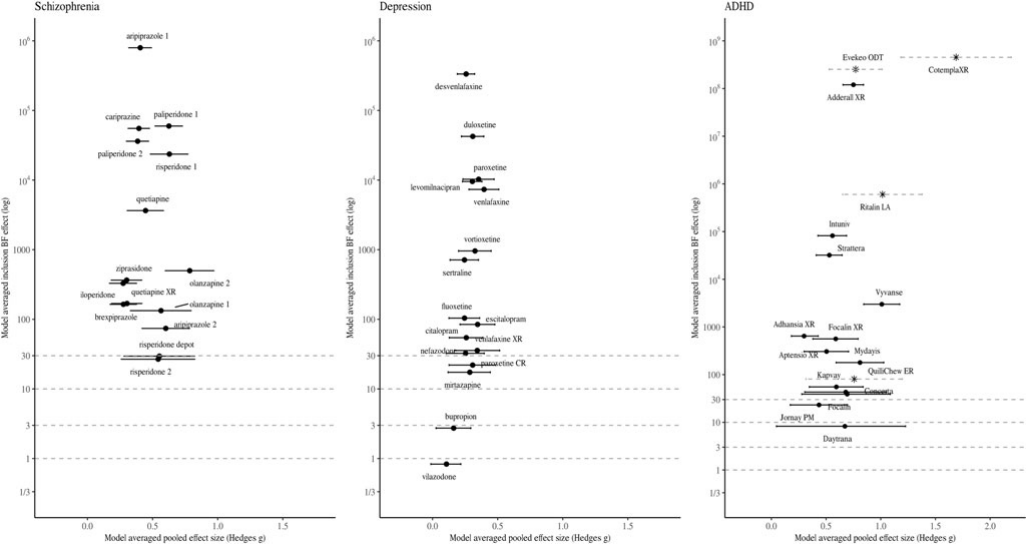
Model-averaged meta-analytic *BF*s and pooled effect estimates. Error bars represent 95% highest density interval. Note that the x- and y-axis has different dimensions for medication approved for ADHD. For some drug BFs and effect size correspond to a single trial (indicated by a Asterix and dotted line depicting the 95% confidence intervals). Numbers are used to differentiate drugs with the same non-proprietary name (1 = Abilify, 2 = Aristada, 3 = Zyprexa, 4 = Zyprexa Relprevv, 5 = Invega, 6 = Invega Sustenna, 7 = Risperdal, 8 = Perseris kit).

**Fig. 2.**
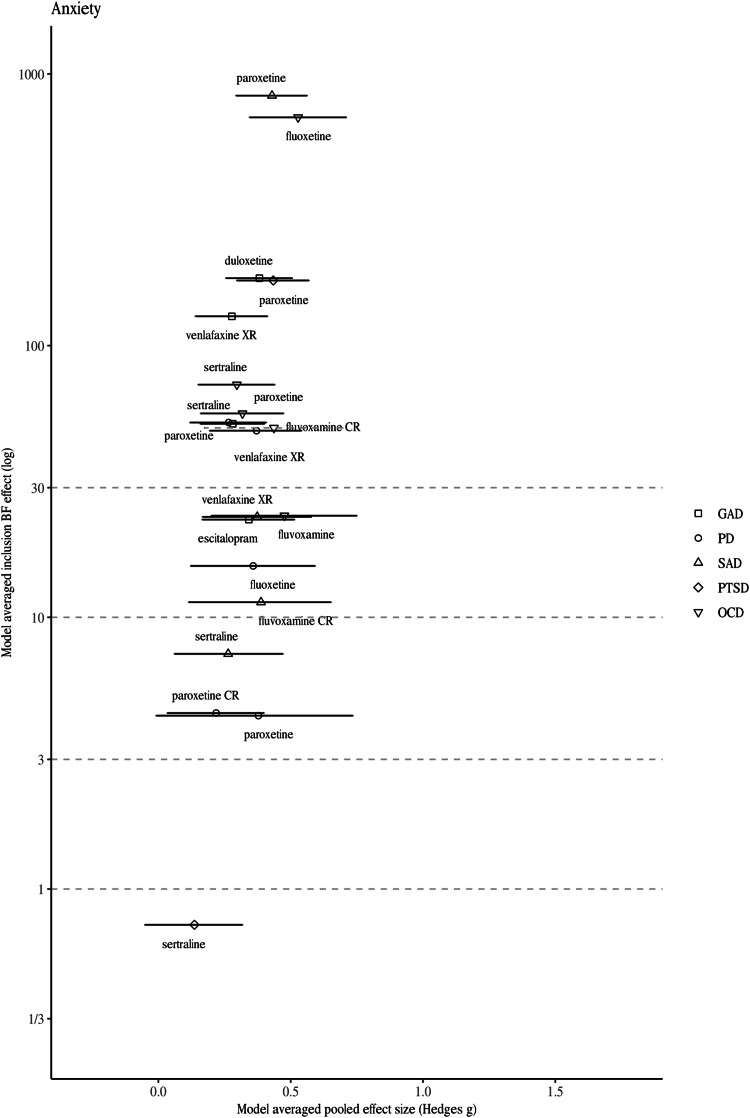
Model-averaged meta-analytic *BF*s and pooled effect estimates for drugs approved for anxiety disorders. Symbols refer to approvals for different indications. Error bars represent 95% highest density interval. For one drug *BF*s and effect size correspond to a single trial (indicated by a dotted line depicting the 95% confidence intervals).

Figures 1 and 2 visualise the results of the BMA meta-analyses for each of the disorder groups. Tables including detailed information for all analyses performed can be found in the online Supplementary material. Overall, three (4.2%) *BF*_BMA_s indicated ambiguous evidence (⅓ < *BF*_BMA_ < 3): sertraline approved for PTSD (*BF*_BMA_ = 0.7), vilazodone (*BF*_BMA_ = 0.5), and bupropion approved for depression (*BF*_BMA_ = 2.7). Four (5.6%) meta-analytic *BF*_BMA_s indicated only modest evidence for a treatment effect (3 < *BF*_BMA_ < 10): Daytrana for ADHD (*BF*_BMA_ = 8.3), sertraline approved for SAD (*BF*_BMA_ = 7.3), and paroxetine (*BF*_BMA_ = 4.4) and paroxetine CR (*BF*_BMA_ = 4.4) for PD. Ten (13.9%) *BF*_BMA_s showed moderately strong evidence for treatment effects (10 < *BF*_BMA_ < 30), including five antidepressants approved for anxiety, two antidepressants for depression, two antipsychotics, and one ADHD medication. The majority of drugs (76.3%) were supported by strong pro-alternative evidence (*BF*_BMA_ > 30).

### Differences in strength of evidence across disorders

Detailed results (i.e. meta-analytic *BF*s and pooled effect sizes, individual trial *BF*s and effect sizes, sample sizes, and number of trials) are presented in online Supplementary Table S1. Summary results are presented in Table 1. Because the distributions of *BF*s were heavily right-skewed, we report the median instead of the mean.

**Table 1.** Overview of meta-analytic *BF*s (*BF*_BMA_) and pooled effect sizes per drug (*ES*_BMA_), individual trial *BF*s (*BF*) and effect sizes (*ES*), sample size for individual trials (*N*_i_), and number of trials (*N*_trials_) across the four disorder groups

		Schizophrenia	Depression	Anxiety	ADHD
*BF*_BMA_	Median	365.4	94.2	49.8	1820.4
	Range (min–max)	26.7–8.0 × 10^5^	0.8–3.3 × 10^5^	0.7–1.3 × 10^5^	8.3–2.3 × 10^15^
*ES*_BMA_	Median	0.45	0.30	0.37	0.72
	Range (min–max)	0.27–0.79	0.11–0.40	0.14–0.55	0.30–1.89
*BF*	Median	21.0	1.3	6.8	185.0
	Range (min–max)	0.1–1.0 × 10^9^	0.1–7.5 × 10^7^	0.1–4.6 × 10^64^	0.2–1.4 × 10^19^
*ES*	Median	0.44	0.27	0.38	0.65
	Range (min–max)	*−*0.13 to 0.92	*−*0.29 to 0.84	*−*0.15 to 1.15	0.04–1.89
*N*_i_	Median	324	218	254	189.5
	Range (min–max)	68–636	29–704	87–565	20–563
*N*_trials_	Median	3	5.5	3	2
	Range (min–max)	1–6	3–16	1–4	1–6

Although the median meta-analytic *BF* of each disorder group indicated ‘very strong evidence’, the strength of evidence differed between disorders. The highest median strength of evidence was found for ADHD (*BF*_BMA_ = 1820.4), followed by antipsychotics for schizophrenia (*BF*_BMA_ = 365.4). Median strength of evidence was lower for antidepressants for depression (*BF*_BMA_ = 94.2) and for anxiety (*BF*_BMA_ = 49.8). Similarly, variability in *BF*_BMA_ differed between disorders. The largest variance was found in ADHD (8.3–2.3 × 10^15^), followed by schizophrenia (26.7–8.0 × 10^5^). For antidepressants approved for depression (0.8–3.3 × 10^5^) and anxiety (0.7–1.3 × 10^5^), the range was the smallest.

### Individual *BF*s and trial characteristics

Median individual trial *BF*s are presented in online Supplementary Table S1 (for a visual representation, see online Supplementary Figs S1 through S4). Individual *BF*s differed (i.e. *BF*s corresponding to individual trials) between the four disorder groups. The median individual *BF* was the highest for ADHD (*BF* = 185.0), followed by antipsychotics (*BF* = 21.0) and anxiety (*BF* = 6.8). The median individual *BF* was the lowest for depression (*BF* = 1.3). Trial strength of evidence varied for all disorder groups (see Table 1).

**Fig. 3.**
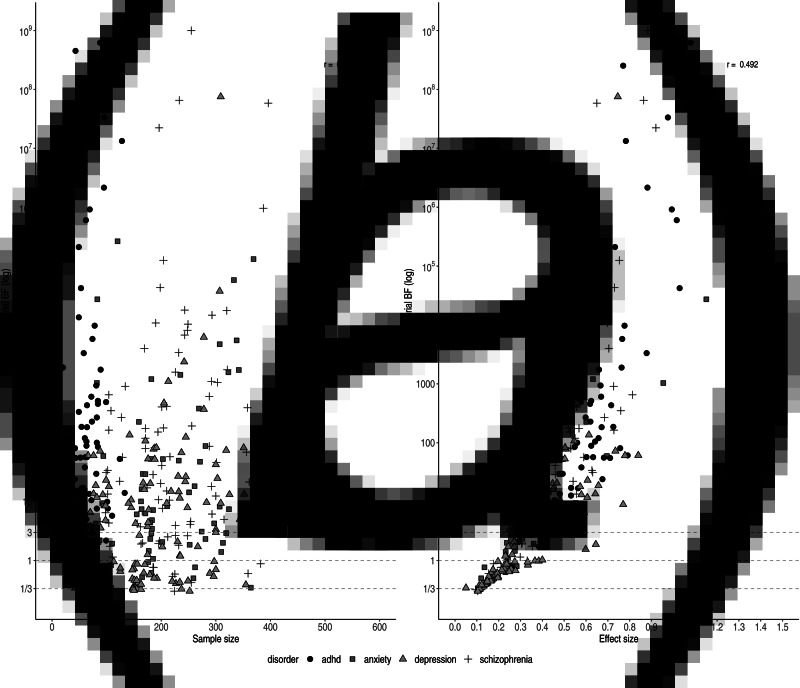
Individual *BF*s on a log scale plotted against sample size (left) and effect size (right)). Symbols and shading indicate the four different disorder groups.

The relationships between individual *BF*s and both effect size and sample size are shown in Fig. 3. The scatterplot of the effect sizes and individual *BF*s shows a positive association (*r*_log(*BF*),*ES*_ = 0.492), whereas the scatterplot of sample sizes and individual *BF*s does not show a clear pattern (e.g. the highest *BF* for ADHD trials had the smallest sample size; *r*_log(*BF*),*N*_ = 0.056), although this may be due to confounding with other factors (such as effect size).

We observed variation in sample size for trials concerning ADHD medication (20–563). This can be partially explained by the inclusion of cross-over trials, which by design have comparatively small sample sizes. Trials were generally randomised, controlled, parallel group trials (parallel-group RCTs); however, crossover trials were sometimes performed for drugs approved for the treatment of ADHD. Smaller sample sizes did not necessarily correspond to ambiguous evidence. For instance, two cross-over trials for ADHD indicated very strong pro-alternative evidence with small sample sizes (*n* = 20, *ES* = 1.74, *BF* = 189.4 and *n* = 39, *BF* = 3.70 × 10^14^). This was the case for parallel trials for depression and schizophrenia, as well (*n* = 66, *ES* = 0.84, *BF* = 61.1 and *n* = 104, *ES* = 0.73, *BF* = 163.7).

The lowest individual *BF*s were found for depression (*BF* = 1.3), which might be explained by the small effect sizes (*ES* = 0.27). As illustrated in Fig. 3*a*, antidepressants for depression commonly displayed the smallest effect sizes, corresponding to the smallest individual *BF*s. In contrast, antipsychotics and ADHD medication showed larger effect sizes (*ES* = 0.44 and *ES* = 0.65, respectively) corresponding to stronger evidential strength.

### Meta-analytic *BF*s and trial characteristics

The very strong evidence obtained for most ADHD medications appeared to be primarily due to high individual *BF*s, as the number of trials for each ADHD drug was very small. In contrast, very strong evidence for depression was generally achieved through a large number of trials, despite small studies and very low individual *BF*s. Larger numbers of trials corresponded to a greater proportion of trials being deemed questionable or negative by the FDA. For example, for paroxetine for depression, 16 trials were mentioned, of which nine were deemed questionable or negative. Our Bayesian re-analysis suggested evidence for an additional trial to be ambiguous. Nonetheless, the meta-analytic *BF* suggested very strong pro-alternative evidence for the drug to treat depression (*BF*_BMA_ = 10 267.8).

**Fig. 4.**
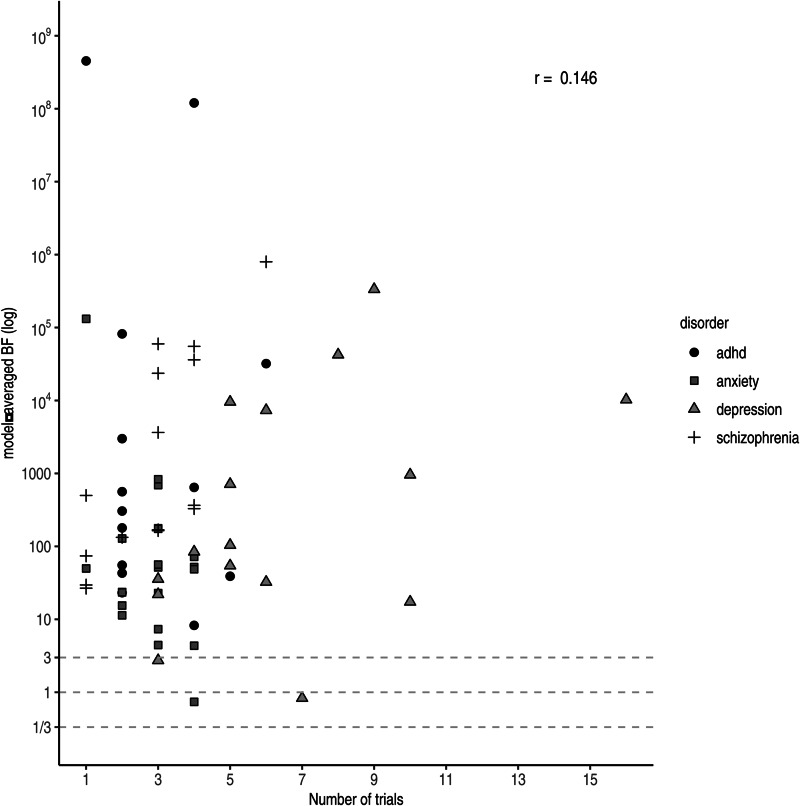
Model-averaged *BF*s on a log scale plotted against the number of performed trials. Symbols and shading indicate the four different disorder groups.

Under a Bayesian framework, more trials (i.e. more data) equal more evidence for the more probable hypothesis. In other words, with accumulating data the evidential strength (i.e. the *BF*) tends to point towards either zero (in case the null hypothesis is true) or infinity (in case the alternative hypothesis is true). However, we do not observe this relationship across drugs in practice (*r*_log(*BF*),Nr. trials_ = 0.146 see Fig. 4). A likely explanation is that more trials are run for drugs with lower effect sizes to compensate. Trials concerning antidepressants approved for anxiety were slightly higher powered (i.e. had larger effect sizes and sample sizes) compared to those for antidepressants approved for depression. Nonetheless, only a few trials were performed per drug resulting in weaker evidence at the drug level compared to the other three disorder groups. For antipsychotics, we observed substantial pro-alternative evidence across the board, while the number of trials was comparable to those of antidepressants for anxiety. However, similar to ADHD medication, the individual studies were on average well-powered (i.e. medium effect size and large sample size), resulting in higher individual *BF*s and consequently stronger evidence at the drug level.

### Sensitivity analysis

Results from the sensitivity analyses are summarised in online Supplementary Table S2. Importantly, the qualitative interpretation did not change for different choices of model and/or scale parameter.

## Discussion

Even though approval of psychotropic drugs is based on the same guideline and processed through the same pathway and by the same group within the FDA, we detected large differences in evidential strength and trial programmes. Although efficacy for the majority of psychotropic drugs was supported by very strong evidence at the time of approval, we observed substantial variation in the strength of evidence between approved psychotropic drugs: ADHD medication was supported by extreme evidence, whereas evidence for antidepressants for both depression and anxiety was considerably lower and more frequently classified as weak or moderate.

Differences in evidential strength might be partly explained by differences in trial programmes. For instance, ADHD drugs typically had very large effect sizes, resulting in extreme evidence for efficacy despite comparatively fewer and smaller trials. All else being equal, larger effect sizes correspond to larger *t* values, which in turn correspond to larger *BF*s. A potential drawback here is that the drug is tested on too few people to effectively gather evidence to rely on for safety. As most ADHD drugs are variants of methylphenidate, this may be considered acceptable. However, one might wonder: if a drug is considered different enough that a new approval application with new trials is needed to establish efficacy, is it reasonable to assume that safety will be the same?

In contrast, for depression in particular we saw clinical trial programmes with comparatively many trials and participants, meaning that there is much more experience with the drug at the time of approval. Evidence for efficacy, however, is considerably lower compared to ADHD and schizophrenia. The most likely explanation for this finding is that effect sizes for antidepressants were generally smaller than for other drug groups. Alternatively, heterogeneous samples for depression and anxiety, due to more ambiguous diagnostic criteria, might have contributed to larger between-study variation and thus lower evidential strength.

Using a Bayesian approach allowed us to identify cases in which psychotropic drugs were approved with moderate or even ambiguous evidence for its efficacy. Approximately, a quarter of all meta-analytic *BF*s fell within this tier (i.e. *BF*_BMA_ < 30). In a few instances, drugs were approved despite ambiguous statistical evidence (i.e. 1/3 < *BF*_BMA_ < 3). Sometimes approval was based on other considerations. For example, bupropion SR (sustained-release) was approved based on bio-equivalence with immediate-release bupropion, despite negative efficacy trials for bupropion SR (U.S. Food and Drug Administration ‘Bupropion SR’, 1996). Other times, negative or ‘failed’ trials were not included in the efficacy determination. For example, for vilazodone, three of five trials were considered ‘failed’, as the active comparator did not separate from placebo. The FDA has a history of ignoring failed trials because they supposedly lack assay sensitivity, the ability to differentiate an effective treatment from a less effective or ineffective one (Chuang-Stein, 2014). Although other considerations certainly play a role in the approval process, the example of vilazodone illustrates how the FDA's current practice of determining efficacy using two independent statistically significant trials (regardless of the number of additional negative trials) *can* lead to inconsistent decision making in practice. Under the Bayesian framework, endorsement of this drug would not have been recommended.

### How *BF*s could aid evidence-based treatment choices

For the purpose of drug development and endorsement, Bayesian meta-analysis offers several advantages over classical, frequentist meta-analysis, suggested by the FDA (U.S. Department of Health and Human Services et al., 2017). Although frequentist meta-analysis is well-equipped to estimate the size of a treatment effect and its uncertainty (van Ravenzwaaij & Ioannidis, 2019), it cannot differentiate between the absence of evidence (uncertainty regarding the effect) and evidence of absence (e.g. evidence for effect = 0; a similar argument was made by Monden et al., 2018). This is especially important in the context of failed or negative trials, which could either indicate insufficient data or non-effectiveness of the drug. If the problem is merely the absence of evidence, the sponsor might perform additional trials to prove efficacy, whereas non-approval should be issued when evidence of absence has been demonstrated.

Bayesian meta-analysis yields *both* pooled effect sizes and evidential strength. Effect size estimates from the current analysis are similar to those from previous meta-analyses. Combining pre- and post-marketing studies, effect sizes for methylphenidate for ADHD, antipsychotics, and antidepressants approved for depression were estimated to be 0.77, 0.51, and 0.38, respectively (Leucht et al., 2015). These estimates are slightly larger than ours (i.e. 0.72, 0.45, and 0.30, respectively), which may be because we included unpublished, negative trials. Moreover, our effect size estimates are similar to previous network meta-analyses, which aimed to compare the efficacy and safety profiles between antipsychotics (Huhn et al., 2019), antidepressants for depression (Cipriani et al., 2018), and ADHD medication (Cortese et al., 2018). For example, estimates for antipsychotics ranged from 0.27 to 0.89 (Huhn et al., 2019; here: 0.27–0.79) and the pooled effect size for antidepressants was 0.30 (Cipriani et al., 2018; here: 0.30).

Our study adds novel information to previous research by using *BF*s to estimate the strength of evidence. The network meta-analyses conclude with rankings based on efficacy and safety data. We offer additional insight into the strength of evidence for efficacy. Sometimes, our rankings align, lending further support to the efficacy of the drug. For example, based on effect size, Huhn et al. (2019) ranked risperidone in the top tier and our analysis additionally indicates very strong support for the treatment effect. In other cases, *BF*s advise caution. For example, based on effect size, Huhn et al. (2019) ranked olanzapine highly, whereas our analysis places it in the lower quarter in comparison with the other drugs. Our analysis suggests that all else being equal, risperidone should be preferred over olanzapine. Additionally, *BF*s can help to refine rankings based on efficacy and safety data. For example, Cipriani et al. (2018) performed a network meta-analysis pooling efficacy and safety data for antidepressants for depression. Based on relatively high response rates and relatively low dropout rates, they recommended – among others – mirtazapine and paroxetine. Here, paroxetine is supported by extreme evidence, whereas mirtazapine has the third lowest evidential strength. All else being equal, paroxetine should be preferred over mirtazapine. For the purpose of drug prescription, *BF*s offer a valuable source of information for clinicians. Prescription and use of psychotropic drugs has steadily increased over the past few decades (Ilyas & Moncrieff, 2012; Olfson & Marcus, 2009; Stephenson, Karanges, & McGregor, 2013). With a wide variety of drugs available, choosing the most appropriate one can be difficult, highlighting the importance of good evidence. Next to safety and patient-specific concerns, considerations regarding effect size and evidential strength play a central role. Commonly, strength of evidence is assessed by qualitative or subjective criteria. The American Psychological Association (APA) considers evidential strength for their recommendations by reviewing the available literature and assessing risk of bias, the degree to which reported effects are unidirectional, directness of the outcome measure, quality of the control condition, and precision of the estimate (e.g. width of a 95% confidence interval; American Psychological Association, 2019; American Psychological Association, 2017). Although these considerations are certainly meaningful, implementing them in clinical practice can be unsystematic, easily influenced by the rater, and might fail to effectively quantify strength of evidence (i.e. the likelihood of the treatment effect existing). For example, the APA recommends sertraline for the treatment of PTSD and argues that this decision is supported by the moderate strength of evidence. In contrast, our analysis suggests no evidence for a treatment effect of sertraline at the time of approval for PTSD.

Moreover, *BF*s offer a valuable source of information when effect sizes are highly comparable between drugs. For instance, the APA concludes that many antidepressants are equally effective (American Psychological Association, 2019) and makes no clear recommendation which one to prefer. In these cases, *BF* could be used as an additional criterion, as antidepressants vary substantially in evidential strength (see also Monden et al., 2018). For example, leaving aside non-efficacy considerations, but considering both the effect size and evidential strength, one might choose venlafaxine or paroxetine over sertraline or citalopram, two very commonly prescribed antidepressants (Moore & Mattison, 2017; although we acknowledge that safety/tolerability considerations may alter this choice). This advantage still holds if effect sizes vary from medium to large. For example, for ADHD drugs, Cotempla XR, Evekeo ODT, and Adderall clearly demonstrated the highest evidential strength with comparable effect size and might be preferred over the others.

### Strengths and limitations

Adopting a Bayesian framework enabled us to capture differences in evidential strength between disorders and drug groups. Nonetheless, the results should be considered in light of a few limitations. First, although we used information from the FDA-registered trials, limiting the influence of reporting bias, we were confined to data from approved drugs and pre-marketing studies. Consequently, we cannot speak to the process and statistical evidence of non-approval, or strength of evidence after post-marketing studies. As such, the current results only reflect the evidential strength at the time of approval but are not necessarily accurate reflections of the current state of evidence. Second, some values were unavailable and had to be imputed, which might have introduced extra noise. Nonetheless, imputation did not seem to be associated with increased between-study heterogeneity as indicated by comparable posterior probabilities of the random-effect model between drugs for which test statistics were available and drugs for which test statistics were imputed. Finally, Bayesian analysis is dependent on the choice of prior. Although this is an often-heard critique, we mostly restricted our analyses to default priors to ensure comparability of our results across drug groups. Furthermore, our sensitivity analysis indicated that different choices for the scale parameter of the prior did not change interpretation of the *BF* qualitatively in the present analysis.

The main strength of our study is that, to our knowledge, we have performed the first large-scale comparison of evidential strength between several disorders. Previously, Bayesian methods have been proposed for and discussed in the context of the drug development and endorsement (Burke, Billingham, Girling, & Riley, 2014; Cipriani et al., 2018; Huhn et al., 2019; Monden et al., 2016, 2018; van Ravenzwaaij & Ioannidis, 2019; Woodcock, Temple, Midthun, Schultz, & Sundlof, 2005). In recent years, Bayesian network meta-analyses specifically became increasingly popular in medical sciences (Hamza et al., 2021; Holper, 2020; Huhn et al., 2019). Our study differs from previous studies that were concerned with efficacy and tolerability, but that either did not address evidential strength or did not compare evidential strength across different psychological disorders. Here, we provided an overview of the evidential standard for psychotropic drugs at the time of FDA approval and demonstrated how psychotropic drugs differ in their evidential strength, using *BF*s.

## Conclusion

Taken together, the present analysis offers interesting insights into the evidential strength within and across different psychotropic drugs. We observed large differences in evidential strength and trialling between disorders. Although the majority of re-analysed drugs was supported by substantial evidence, we also observed cases where the current approval process led to endorsement despite ambiguous statistical evidence. Moreover, evidential strength differed greatly between drugs and across disorder groups. Lower evidential support for efficacy was observed more frequently for antidepressants. Differences in evidential strength might be a consequence of different standards in trialling. The *BF* as a measure of evidential strength might offer a valuable, additional source of information and helps to set up a consistent and transparent standard for evaluating strength of evidence of efficacy in the approval process of psychotropic drugs.

## Data Availability

The datasets generated and analysed during the current study are available at OSF, https://osf.io/364t5.

## Footnotes

† The notes appear after the main text.

## Notes

1 For reporting of ADHD drugs, we use the commercial instead of the non-proprietary names as they are common variants of the same active agent.

2 That is, fluvoxamine CR approved for OCD, paroxetine CR approved for SAD, and seven drugs approved for the treatment of ADHD (i.e. Contempla XR, Daynavel XR, Evekeo ODT, Metadate CD, QuilliChew, Quillivant, and Ritalin LA).

## Supplementary material

For supplementary material accompanying this paper visit https://doi.org/10.1017/S00332917210039508. click here to view supplementary material
