# Supplementary material for: Comparing the evidential strength for psychotropic drugs: a Bayesian meta-analysis
Source: Psychol Med. 2021 Oct 8;51(16):2752–61. doi: 10.1017/S0033291721003950 (PMC8640368; doi:10.1017/S0033291721003950)
Supplement: Supplementary file 1 [file S0033291721003950sup001.docx]

Supplement:

**Comparing the Evidential Strength for Psychotropic Drugs: A Bayesian Meta-Analysis**

Table of Contents

[Background 2](#_Toc80975249)

[Justification Bayesian model-averaging. 2](#_Toc80975250)

[Method 3](#_Toc80975251)

[Data from FDA reviews. 3](#_Toc80975252)

[Strategies when no precise p-value was available 3](#_Toc80975253)

[Formulas 4](#_Toc80975254)

[Deviation from Preregistration 5](#_Toc80975255)

[Sensitivity Analysis 5](#_Toc80975256)

[Results 7](#_Toc80975257)

[Individual Trial *BFs*. 7](#_Toc80975258)

[Visual presentation of individual Bayes Factors 22](#_Toc80975259)

[Meta-analytic Bayes Factors 26](#_Toc80975260)

[Sensitivity Analysis 29](#_Toc80975261)

[References 30](#_Toc80975262)

# Background

## Justification Bayesian model-averaging.

In the article *Comparing the Evidential Strength for Psychotropic Drugs: A Bayesian Meta-Analysis* we implemented Bayesian model averaging to combine a fixed-effect and random-effect model. This method fully acknowledges uncertainty with respect to the choice between a random or fixed-effect model (Gronau et al., 2017). A fixed-effects model makes the assumption that all studies have the same underlying “true” effect size, meaning that variation between studies is solely due to sampling error (Gronau et al., 2017; Rice et al., 2018). A random-effect model allows for idiosyncratic study effects (Gronau et al., 2017) by including an additional error term representing variation between studies (Borenstein et al., 2009). As such, the random model is more complex than the fixed-effects model (Scheibehenne et al., 2017). Commonly, the default recommendation in the meta-analysis literature is to adopt random-effect meta-analysis, as the fixed-effect assumption is unlikely to hold (e.g. Borenstein et al., 2010). However, this notion has been challenged recently (we refer the interested reader to Rice et al., 2018). Here, we will be dealing with the situation where we are combining a limited number of trials per drug. From previous work (i.e., Monden et al., 2016, 2018; Turner et al., 2008, 2012), we expect the number of trials per drug to vary between 1 and 10. In these situations, the more complex random-effect model might overfit the data (Scheibehenne et al., 2017). Thus, instead of adopting one model only and ignoring the other one, we will adopt an approach put forward by Gronau and colleagues (2017) and weight the results from both models according to their posterior plausibility.

# Method

## Data from FDA reviews.

Data on antidepressants approved for the treatment of depression between 1987 and 2016 were first extracted by Turner et al (Turner et al., 2008). and later updated by de Vries et al. (De Vries et al., 2018). Data on antidepressants approved for the treatment of anxiety disorder between 1998 and 2008 were extracted by de Vries et al. (de Vries et al., 2016) and Roest et al.(Roest et al., 2015). Data on antipsychotics approved for the treatment of schizophrenia between 1993 and 2018 were extracted by Turner et al. (Turner et al., 2013) and updated by the first two authors of this manuscript (MMP and YAdV). Data for medication approved for the treatment of ADHD between 2000 and 2019 were extracted by MMP and YAdV.

For each drug, the corresponding FDA reviews were retrieved from the FDA’s website https://www.accessdata.fda.gov/scripts/cder/daf/index.cfm. If reviews were not publicly available, they were requested from the FDA’s Freedom of Information Office (Turner et al., 2013). Within the Drug Approval package, data relevant to FDA’s determination of drug efficacy were examined.

Efficacy data on all phase 2 and 3 clinical trials were extracted preferably from the statistical review and from the medical review or team leader memos, if necessary. Trials were generally randomized, controlled, parallel group trials (parallel-group RCTs); however, crossover trials were sometimes performed for drugs approved for the treatment of ADHD.

## Strategies when no precise p-value was available

If exact *p*-values were not provided, we used: (1) the mean difference between drug and placebo group and associated standard error, SE (or standard deviation, SD, or confidence interval, CI); (2) the mean change in drug and placebo group (with SE/SD/CI); or (3) the mean endpoint score for the drug and placebo group (with SE/SD/CI). If none of these statistics were available, we (4) sampled *t*-statistics from an aggregated distribution of *t*-values constructed from the available data in that specific disorder group. For a particular missing value, the distribution was truncated based on the available, non-exact *p*-value. See the supplementary RMarkdown file for details of the procedure. We sampled nine *t*-statistics per case to ensure robustness.

For cross-over trials, we used: (1) mean differences and associated SEs or SDs; or (2) mean differences and associated 95%CI to calculate a paired *t*-test. If no information regarding the SE and/or SDs of within-participant differences was available, (3) a correlation coefficient was used to impute the values (Higgins et al., 2019). The correlation was calculated from the other cross-over studies, for which all necessary information was available. Lastly, if none of the approaches was feasible, we (4) sampled a *t*-value from an aggregated distribution of *t*-values similar to the approach mentioned above.

## Formulas

For cross-over studies, a correlation coefficient was used to impute within-participant differences. The correlation was calculated from the other cross-over studies, for which all necessary information was available using:

| $corr= \frac{{SD}_{1}^{2}+{SD}_{2}^{2}-{SD}_{diff}^{2}}{2* {SD}_{1}*{SD}_{2}}$ | (1) |
| --- | --- |

For parallel group trials, we calculated a corrected Hedges g using the following:

| $g=\left( t*\sqrt{\frac{1}{n_{1}}+\frac{1}{n_{2}}} \right)*1- \frac{3}{4(n_{1}+n_{2}-1)}$ | (2) |
| --- | --- |

with n_1_ being the sample size of the intervention group and n_2_ being the sample size of the control group.

For cross-over trials, we calculated the uncorrected standardized mean difference (SMD) using:

|  | $SMD= \frac{MD}{{SD}_{pooled}}$ | (3) |
| --- | --- | --- |
| where | ${SD}_{pooled}= \sqrt{\frac{{SD}_{1}^{2}+{SD}_{2}^{2}}{2}}$ | (4) |
| and | $SE\left( SMD \right)\sqrt{\frac{1}{N}+\frac{{SMD}^{2}}{2N}}*\sqrt{2(1-corr)}$ | (5) |

## Deviation from Preregistration

During data-analysis, we made three additional choices that were not preregistered. First, we adjusted calculation of the effect size for cross-over trials to account for dependency of the groups. Second, in the anxiety data-set, we encountered a few trials for which calculations were based on approximated *p*-values. For trials concerning panic disorder, which frequently employed dichotomous outcomes, we had additional information (i.e., proportion of participants without panic attacks) to calculate a test statistic with associated effect size. We chose to calculate chi-square statistics based on contingency tables instead. To calculate chi-squares for contingency tables, we used the [chisq.test](https://www.rdocumentation.org/packages/stats/versions/3.6.2/topics/chisq.test) function in R to calculate the test statistic. The SMD was calculated by:

| $SMD=LogOddsRatio*\frac{\sqrt{3}}{\pi}$ | (6) |
| --- | --- |

Third, for two trials (i.e., FDA trial number TRP-BP-EF002 and 3002) in the ADHD data-set, the FDA reviews only reported adjusted *p*-values. Thus, we utilized other information to calculate the *t*-value.

## Sensitivity Analysis

We included a sensitivity analysis as a robustness check of various choices that we made regarding our data analysis. First, we examined the difference in estimated pooled effect sizes and meta-analytic *BF*s depending on the choice of model (i.e., fixed vs. random). Second, we performed sensitivity analyses regarding our choice of priors. Following Monden et al. (Monden et al., 2016, 2018), we calculated *BF*s using a “skeptical” prior with $r=\frac{1}{6}*\sqrt{2}$ and an “optimistic” prior with $r=\frac{2}{3}*\sqrt{2}$. Third, we performed sensitivity analyses comparing the estimated effects with and without imputed values. Fourth, we performed sensitivity analyses regarding the inclusion of cross-over trials. Analysis was performed excluding cross-over trials and estimated effect sizes and evidence load were compared to the analyses including cross-over trials. Fifth, we performed one additional, not preregistered, sensitivity analysis where we first combined all dosages within a trial using a fixed-effects meta-analysis before performing model-averaged Bayesian meta-analysis using the pooled estimates.

# Results

## Individual Trial *BFs*.

Table S1 summarizes all trials included in this Bayesian meta-analytic review. Trials considered questionable or failed by the FDA are highlighted in light gray, trials considered negative are highlighted in dark gray.

| Table S1  *Summary of the analyzed FDA trials, p-values, and individual trial Bayes factors* | | | | | | | |
| --- | --- | --- | --- | --- | --- | --- | --- |
| Drug | Trial Number | | Dose (mg/d) | Sample size | Effect size | *p* | *BF* |
| ***Schizophrenia*** |  | |  |  |  |  |  |
| Aripiprazole | 93202 | | placebo | 35 |  |  |  |
| (Abilify) |  | | 30 | 33 | 0.33 | .173 | 1.00 |
|  | 97201 | | placebo | 102 |  |  |  |
|  |  | | 15 | 99 | 0.56 | .0001 | 405.09 |
|  |  | | 30 | 100 | 0.37 | .0089 | 7.72 |
|  | 97202 | | placebo | 103 |  |  |  |
|  |  | | 20 | 98 | 0.46 | .0013 | 41.01 |
|  |  | | 30 | 96 | 0.43 | .0029 | 20.38 |
|  | 138001 | | placebo | 107 |  |  |  |
|  |  | | 10 | 103 | 0.55 | <.001 | 416.09 |
|  |  | | 15 | 103 | 0.40 | .004 | 15.18 |
|  |  | | 20 | 97 | 0.52 | <.001 | 178.44 |
|  | 94202 | | placebo | 57 |  |  |  |
|  |  | | 10 | 51 | 0.03 | .894 | 0.23 |
|  |  | | 30 | 54 | 0.30 | .117 | 1.15 |
| Aripiprazole | 31-03-239 | | placebo | 98 |  |  |  |
| (Abilify) |  | | 10 | 99 | 0.29 | .041 | 2.14 |
|  |  | | 30 | 97 | 0.40 | .006 | 10.78 |
| Aripiprazole | ALK9097-003 | | placebo | 194 |  |  |  |
| (Aristada) |  | | 441 | 193 | 0.58 | <.001 | 9.59*10^5^ |
|  |  | | 882 | 202 | 0.65 | <.001 | 5.84*10^7^ |
| Brexpiprazole | 230 | | placebo | 180 |  |  |  |
|  |  | | 2 | 179 | 0.15 | .145 | 0.60 |
|  |  | | 4 | 181 | 0.32 | .002 | 21.55 |
|  | 231 | | placebo | 178 |  |  |  |
|  |  | | 2 | 180 | 0.42 | <.0001 | 392.98 |
|  |  | | 4 | 178 | 0.37 | .0006 | 69.40 |
| (continued) | | | | | | | |
| Table 1. (continued) | | | | | | | |
| Drug | Trial Number | | Dose (mg/d) | Sample size | Effect size | *p* | *BF* |
|  | 203 | | placebo | 93 |  |  |  |
|  |  | | 2-3 | 90 | 0.08 | .584 | 0.26 |
|  |  | | 4-6 | 92 | 0.21 | .151 | 0.77 |
| Cariprazine | RGH-MD-04 | | placebo | 149 |  |  |  |
|  |  | | 3 | 151 | 0.33 | .0044 | 12.46 |
|  |  | | 6 | 154 | 0.48 | <.0001 | 1055.96 |
|  | RGH-MD-16 | | placebo | 148 |  |  |  |
|  |  | | 1.5 | 140 | 0.41 | .0005 | 87.18 |
|  |  | | 3 | 140 | 0.48 | <.0001 | 611.91 |
|  |  | | 4.5 | 148 | 0.57 | <.0001 | 14695.99 |
|  | RGH-MD-05 | | placebo | 145 |  |  |  |
|  |  | | 3-6 | 147 | 0.35 | .0029 | 18.13 |
|  |  | | 6-9 | 147 | 0.49 | <.0001 | 1105.18 |
|  | RGH-MD-03 | | placebo | 126 |  |  |  |
|  |  | | 1.5-4.5 | 122 | 0.28 | <.05 | 2.63 |
|  |  | | 6-12 | 129 | 0.14 | >.05 | 0.42 |
| Iloperidone | 3101 | | placebo | 140 |  |  |  |
|  |  | | 24 | 283 | 0.28 | .007 | 7.65 |
|  | 3000 | | placebo | 117 |  |  |  |
|  |  | | 12 | 115 | 0.26 | .047 | 1.82 |
|  | 3004 | | placebo | 152 |  |  |  |
|  |  | | 10-16 | 149 | 0.38 | .001 | 46.21 |
|  | 3005 | | placebo | 152 |  |  |  |
|  |  | | 12-16 | 230 | 0.18 | .09 | 0.88 |
|  |  | | 20-24 | 141 | 0.30 | .01 | 6.16 |
| Olanzapine | HGAD | | placebo | 62 |  |  |  |
| (Zyprexa) |  | | 10 | 62 | 0.54 | .003 | 22.21 |
|  |  | | 15 | 65 | 0.67 | <.001 | 269.00 |
|  | HGAP | | placebo | 49 |  |  |  |
|  |  | | 10 | 49 | 0.50 | .014 | 6.46 |
| Olanzapine | HGJZ | | placebo | 98 |  |  |  |
| (Zyprexa relprevv) |  | | 210 mg/2weeks | 106 | 0.75 | <.0001 | 124953.51 |
|  |  | | 300 mg/2weeks | 98 | 0.92 | <.0001 | 2.22*10^7^ |
|  |  | | 405 mg/2weeks | 100 | 0.73 | <.0001 | 43071.00 |
| Paliperidone | 303 | | placebo | 126 |  |  |  |
| (Invega) |  | | 6 | 123 | 0.61 | <.001 | 10331.41 |
|  |  | | 9 | 122 | 0.60 | <.001 | 8114.33 |
|  |  | | 12 | 129 | 0.88 | <.001 | 9.97*10^8^ |
|  | 304 | | placebo | 105 |  |  |  |
|  |  | | 6 | 110 | 0.38 | .006 | 10.61 |
|  |  | | 12 | 111 | 0.48 | <.001 | 57.86 |
|  | 305 | | placebo | 120 |  |  |  |
|  |  | | 6 | 123 | 0.60 | <.001 | 6740.24 |
|  |  | | 9 | 123 | 0.63 | <.001 | 17895.31 |
|  |  | | 15 | 113 | 0.86 | <.001 | 6.54*10^7^ |
| (continued) | | | | | | | |
| Table 1. (continued) | | | | | | | |
| Drug | Trial Number | | Dose (mg/d) | Sample size | Effect size | *p* | *BF* |
| Paliperidone | PSY-3007 | | placebo | 160 |  |  |  |
| (Invega sustenna) |  | | 25 | 155 | 0.28 | .0124 | 4.98 |
|  |  | | 100 | 161 | 0.48 | <.001 | 1734.48 |
|  |  | | 150 | 160 | 0.55 | <.001 | 17484.73 |
|  | PSY-3003 | | placebo | 132 |  |  |  |
|  |  | | 50 | 93 | 0.18 | .193 | 0.59 |
|  |  | | 100 | 94 | 0.32 | .019 | 3.94 |
|  |  | | 150 | 30 | 0.07 | not tested | 0.28 |
|  | PSY-3004 | | placebo | 125 |  |  |  |
|  |  | | 25 | 129 | 0.31 | .015 | 4.58 |
|  |  | | 50 | 128 | 0.30 | .017 | 4.12 |
|  |  | | 100 | 131 | 0.47 | <.001 | 192.64 |
|  | SCH-201 | | placebo | 66 |  |  |  |
|  |  | | 50 | 63 | 0.59 | .001 | 56.78 |
|  |  | | 100 | 68 | 0.73 | <.0001 | 907.74 |
| Quetiapine | 0001/0008 | | placebo | 94 |  |  |  |
|  |  | | ≤ 250 | 92 | 0.21 | .151 | 0.77 |
|  |  | | ≤ 750 | 94 | 0.51 | .0006 | 82.50 |
|  | 0006 | | placebo | 53 |  |  |  |
|  |  | | 75-750 | 53 | 0.35 | .0694 | 1.75 |
|  | 0013 | | placebo | 51 |  |  |  |
|  |  | | 150 | 48 | 0.63 | .002 | 31.37 |
|  |  | | 300 | 51 | 0.63 | .0019 | 33.99 |
|  |  | | 600 | 51 | 0.56 | .0057 | 13.45 |
|  |  | | 750 | 53 | 0.45 | .0224 | 4.34 |
| Quetiapine XR | 132 | | placebo | 115 |  |  |  |
|  |  | | 400 | 111 | 0.29 | .03 | 2.66 |
|  |  | | 600 | 111 | 0.58 | <.0001 | 1584.08 |
|  |  | | 800 | 117 | 0.59 | <.0001 | 3384.02 |
|  | 41 | | placebo | 78 |  |  |  |
|  |  | | 300 | 83 | -0.01 | .952 | 0.16 |
|  |  | | 600 | 87 | 0.40 | .011 | 6.87 |
|  |  | | 800 | 85 | 0.31 | .052 | 1.91 |
|  | 133 | | placebo | 111 |  |  |  |
|  |  | | 300 | 113 | 0.101 | .434 | 0.30 |
|  |  | | 600 | 101 | 0.29 | .033 | 2.52 |
|  |  | | 800 | 110 | 0.17 | .214 | 0.54 |
| Risperidone | 201 | | placebo | 53 |  |  |  |
| (Risperdal) |  | | 1-10 | 51 | 0.73 | <.001 | 163.74 |
|  | 204-US sites | | placebo | 86 |  |  |  |
|  |  | | 6 | 85 | 0.55 | <.001 | 119.58 |
|  |  | | 10 | 85 | 0.57 | <.001 | 175.31 |
|  |  | | 16 | 85 | 0.53 | <.001 | 79.99 |
|  | SCH-302 | | placebo | 54 |  |  |  |
|  |  | | 1-3 | 54 | 0.76 | <.001 | 348.97 |
|  |  | | 4-6 | 50 | 0.81 | <.001 | 651.64 |
| (continued) | | | | | | | |
| Table 1. (continued) | | | | | | | |
| Drug | Trial Number | | Dose (mg/d) | Sample size | Effect size | *p* | *BF* |
| Risperidone | 09-0010 | | placebo | 84 |  |  |  |
| (Perseris kit) |  | | 90 | 90 | 0.44 | .0037 | 17.12 |
|  |  | | 120 | 85 | 0.70 | <.0001 | 3984.73 |
| Risperidone depot | Ris-USA-121 | | placebo | 92 |  |  |  |
|  |  | | 25 | 93 | 0.46 | .002 | 28.72 |
|  |  | | 50 | 98 | 0.70 | <.001 | 10805.01 |
| Ziprasidone | 104 | | placebo | 47 |  |  |  |
|  |  | | 40 | 55 | 0.18 | .354 | 0.50 |
|  |  | | 80 | 47 | -0.13 | .535 | 0.14 |
|  | 106 | | placebo | 47 |  |  |  |
|  |  | | 40 | 43 | 0.09 | .657 | 0.32 |
|  |  | | 120 | 41 | 0.49 | .022 | 4.61 |
|  | 114 | | placebo | 91 |  |  |  |
|  |  | | 80 | 104 | 0.29 | .047 | 1.94 |
|  |  | | 160 | 103 | 0.53 | .0003 | 152.01 |
|  | 115 | | placebo | 80 |  |  |  |
|  |  | | 40 | 86 | 0.31 | .049 | 1.99 |
|  |  | | 120 | 76 | 0.37 | .02 | 4.24 |
|  |  | | 200 | 82 | 0.36 | .023 | 3.73 |
| ***Depression*** |  | |  |  |  |  |  |
| Bupropion | 203 | | placebo | 117 |  |  |  |
|  |  | | 300 | 113 | 0.27 | .04 | 2.08 |
|  | 205 | | placebo | 116 |  |  |  |
|  |  | | 300 | 111 | 0.08 | .530 | 0.25 |
|  |  | | 400 | 111 | 0.14 | .300 | 0.41 |
|  | 212 | | placebo | 145 |  |  |  |
|  |  | | 300 | 144 | 0.17 | .16 | 0.61 |
| Citalopram | 85A | | placebo | 82 |  |  |  |
|  |  | | 20-80 | 78 | 0.34 | .034 | 2.69 |
|  | 91206 | | placebo | 124 |  |  |  |
|  |  | | 40 | 120 | 0.39 | .0025 | 21.86 |
|  |  | | 60 | 110 | 0.37 | .0053 | 11.51 |
|  | 86141 | | placebo | 50 |  |  |  |
|  |  | | 10-30 | 97 | 0.17 | .316 | 0.49 |
|  | 89303 | | placebo | 64 |  |  |  |
|  |  | | 40 | 61 | 0.22 | .224 | 0.66 |
|  | 89306 | | placebo | 88 |  |  |  |
|  |  | | 40 | 97 | 0.01 | .964 | 0.17 |
| Desvenlafaxine | 332 | | placebo | 150 |  |  |  |
|  |  | | 50 | 150 | 0.27 | .02 | 3.37 |
|  |  | | 100 | 147 | 0.20 | .09 | 0.96 |
|  | 223 | | placebo | 78 |  |  |  |
|  |  | | 200 | 63 | 0.09 | .59 | 0.29 |
|  |  | | 400 | 72 | 0.10 | .52 | 0.31 |
| (continued) | | | | | | | |
| Table 1. (continued) | | | | | | | |
| Drug | Trial Number | | Dose (mg/d) | Sample size | Effect size | *p* | *BF* |
|  | 306 | | placebo | 118 |  |  |  |
|  |  | | 100 | 114 | 0.38 | .004 | 14.72 |
|  |  | | 200 | 116 | 0.23 | .076 | 1.22 |
|  |  | | 400 | 113 | 0.41 | .002 | 27.02 |
|  | 308 | | placebo | 124 |  |  |  |
|  |  | | 200 | 121 | 0.40 | .002 | 26.56 |
|  |  | | 400 | 124 | 0.34 | .008 | 7.91 |
|  | 304 | | placebo | 114 |  |  |  |
|  |  | | 100-200 | 120 | 0.14 | .277 | 0.43 |
|  | 309 | | placebo | 120 |  |  |  |
|  |  | | 200-400 | 117 | 0.11 | .381 | 0.33 |
|  | 317 | | placebo | 125 |  |  |  |
|  |  | | 200-400 | 110 | 0.09 | .488 | 0.27 |
|  | 320 | | placebo | 118 |  |  |  |
|  |  | | 200-400 | 117 | 0.23 | .078 | 1.19 |
|  | 333 | | placebo | 161 |  |  |  |
|  |  | | 50 | 164 | 0.32 | .004 | 13.18 |
|  |  | | 100 | 158 | 0.39 | NA | 74.34 |
| Duloxetine | HMAT-B | | placebo | 89 |  |  |  |
|  |  | | 40 | 86 | 0.35 | .022 | 3.77 |
|  |  | | 80 | 91 | 0.45 | .003 | 20.32 |
|  | HMAY-A | | placebo | 93 |  |  |  |
|  |  | | 80 | 95 | 0.49 | .001 | 52.48 |
|  |  | | 120 | 93 | 0.51 | <.001 | 78.31 |
|  | HMBH-A | | placebo | 122 |  |  |  |
|  |  | | 60 | 123 | 0.46 | <.001 | 124.91 |
|  | HMBH-B | | placebo | 139 |  |  |  |
|  |  | | 60 | 128 | 0.24 | .047 | 1.72 |
|  | HMAQ-A | | placebo | 57 |  |  |  |
|  |  | | 20-60 | 56 | 0.27 | .146 | 0.95 |
|  | HMAY-B | | placebo | 99 |  |  |  |
|  |  | | 80 | 93 | 0.16 | .253 | 0.50 |
|  |  | | 120 | 103 | 0.27 | .054 | 1.71 |
|  | HMAQ-B | | placebo | 72 |  |  |  |
|  |  | | 20-60 | 81 | 0.07 | .681 | 0.25 |
|  | HMAT-A | | placebo | 89 |  |  |  |
|  |  | | 40 | 90 | 0.18 | .222 | 0.57 |
|  |  | | 80 | 81 | 0.23 | .138 | 0.86 |
| Escitalopram | 99001 | | placebo | 189 |  |  |  |
|  |  | | 10 | 188 | 0.28 | .007 | 7.64 |
|  | 99003 | | placebo | 154 |  |  |  |
|  |  | | 10-20 | 155 | 0.31 | .006 | 9.40 |
|  | SCT-MD-01 | | placebo | 119 |  |  |  |
|  |  | | 10 | 118 | 0.44 | .0007 | 67.97 |
|  |  | | 20 | 123 | 0.57 | <.0001 | 2368.14 |
|  | SCT-MD-02 | | placebo | 125 |  |  |  |
|  |  | | 10-20 | 124 | 0.15 | .251 | 0.45 |
| (continued) | | | | | | | |
| Table 1. (continued) | | | | | | | |
| Drug | Trial Number | | Dose (mg/d) | Sample size | Effect size | *p* | *BF* |
| Fluoxetine | 19 | | placebo | 24 |  |  |  |
|  |  | | 40-80 | 22 | 0.77 | .011 | 8.94 |
|  | 27 | | placebo | 163 |  |  |  |
|  |  | | 40-80 | 181 | 0.27 | .012 | 4.96 |
|  | 62-a | | placebo | 56 |  |  |  |
|  |  | | 20 | 103 | 0.13 | .43 | 0.37 |
|  |  | | 40 | 99 | 0.11 | .5 | 0.33 |
|  |  | | 60 | 107 | 0.11 | .5 | 0.32 |
|  | 62-b | | placebo | 48 |  |  |  |
|  |  | | 20 | 97 | 0.48 | .007 | 10.70 |
|  |  | | 40 | 97 | 0.46 | .01 | 7.94 |
|  |  | | 60 | 103 | 0.17 | .34 | 0.46 |
|  | 25 | | placebo | 24 |  |  |  |
|  |  | | 40-80 | 18 | -0.21 | .5 | 0.20 |
| Levomilnacipran | LVM-MD-01 | | placebo | 175 |  |  |  |
|  |  | | 40 | 176 | 0.25 | .0186 | 3.37 |
|  |  | | 80 | 177 | 0.31 | .0038 | 13.40 |
|  |  | | 120 | 176 | 0.37 | .0005 | 82.16 |
|  | LVM-MD-10 | | placebo | 185 |  |  |  |
|  |  | | 40 | 185 | 0.31 | .0027 | 17.81 |
|  |  | | 80 | 187 | 0.30 | .0043 | 11.78 |
|  | LVM-MD-03 | | placebo | 214 |  |  |  |
|  |  | | 40-120 | 215 | 0.27 | .0051 | 9.61 |
|  | LVM-MD-02 | | placebo | 181 |  |  |  |
|  |  | | 40-120 | 174 | 0.12 | .2492 | 0.39 |
|  | F02695 LP2 02 | | placebo | 277 |  |  |  |
|  |  | | 75-100 | 267 | 0.46 | NA | 134983.15 |
| Mirtazapine | 003-020/3220 | | placebo | 39 |  |  |  |
|  |  | | 5-35 | 41 | 0.66 | .004 | 18.82 |
|  | 003-002 | | placebo | 44 |  |  |  |
|  |  | | 5-35 | 44 | 0.73 | .0008 | 72.31 |
|  | 003-022/3220 | | placebo | 50 |  |  |  |
|  |  | | 10-35 | 49 | 0.61 | .003 | 23.14 |
|  | 003-023/3220 | | placebo | 49 |  |  |  |
|  |  | | 5-35 | 49 | 0.47 | .02 | 4.83 |
|  | 003-024-3220 | | placebo | 48 |  |  |  |
|  |  | | 5-35 | 50 | 0.53 | .01 | 8.51 |
|  | 85027 | | placebo | 61 |  |  |  |
|  |  | | 20-60 | 64 | 0.23 | .19 | 0.75 |
|  | 84023 | | placebo | 45 |  |  |  |
|  |  | | 15-50 | 45 | 0.20 | .347 | 0.53 |
|  | 003-021/3220 | | placebo | 48 |  |  |  |
|  |  | | 10-35 | 45 | 0.25 | .22 | 0.75 |
|  | 003-003 | | placebo | 45 |  |  |  |
|  |  | | 10-35 | 45 | 0.14 | .49 | 0.40 |
| (continued) | | | | | | | |
| Table 1. (continued) | | | | | | | |
| Drug | Trial Number | | Dose (mg/d) | Sample size | Effect size | *p* | *BF* |
|  | 003-008 | | placebo | 2 |  |  |  |
|  |  | | 15 | 30 | -0.26 | .318 | 0.15 |
|  |  | | 30 | 28 | -0.29 | .278 | 0.14 |
|  |  | | 60 | 30 | -0.19 | .458 | 0.17 |
| Nefazodone | 03AOA-003 | | placebo | 45 |  |  |  |
|  |  | | 100-500 | 44 | 0.46 | .03 | 3.58 |
|  | 03AOA-004B | | placebo | 75 |  |  |  |
|  |  | | 300-600 | 78 | 0.38 | .02 | 4.26 |
|  | CN104-005 | | placebo | 91 |  |  |  |
|  |  | | 100-600 | 86 | 0.39 | .01 | 7.28 |
|  | CN104-006 | | placebo | 78 |  |  |  |
|  |  | | 100-600 | 80 | 0.15 | .35 | 0.42 |
|  | 03A2-007 | | placebo | 47 |  |  |  |
|  |  | | 300 | 41 | 0.11 | .6 | 0.35 |
|  | 03AOA-004A | | placebo | 77 |  |  |  |
|  |  | | 300-600 | 76 | 0.07 | .66 | 0.25 |
| Paroxetine | 02-001 | | placebo | 53 |  |  |  |
|  |  | | 10-50 | 51 | 0.57 | .004 | 18.01 |
|  | 02-002 | | placebo | 34 |  |  |  |
|  |  | | 10-50 | 36 | 0.59 | .015 | 6.57 |
|  | 02-004 | | placebo | 32 |  |  |  |
|  |  | | 10-50 | 34 | 0.84 | .001 | 61.09 |
|  | 03-001 | | placebo | 37 |  |  |  |
|  |  | | 10-50 | 39 | 0.64 | .006 | 13.58 |
|  | 03-004 | | placebo | 37 |  |  |  |
|  |  | | 10-50 | 37 | 0.48 | .04 | 2.99 |
|  | 03-005 | | placebo | 42 |  |  |  |
|  |  | | 10-50 | 40 | 0.61 | .007 | 11.81 |
|  | 03-006 | | placebo | 37 |  |  |  |
|  |  | | 10-50 | 39 | 0.78 | .001 | 60.54 |
|  | 03-002 | | placebo | 40 |  |  |  |
|  |  | | 10-50 | 40 | 0.26 | .25 | 0.71 |
|  | 03-003 | | placebo | 42 |  |  |  |
|  |  | | 10-50 | 39 | 0.01 | .98 | 0.24 |
|  | 02-003 | | placebo | 33 |  |  |  |
|  |  | | 10-50 | 33 | 0.25 | .311 | 0.65 |
|  | 01-001 | | placebo | 24 |  |  |  |
|  |  | | 10-50 | 24 | 0.37 | .204 | 0.99 |
|  | 07 | | placebo | 12 |  |  |  |
|  |  | | 20 | 13 | 0.60 | >.05 | 1.55 |
|  | 09 | | placebo | 51 |  |  |  |
|  |  | | 20 | 104 | 0.16 | >.05 | 0.46 |
|  |  | | 30 | 99 | 0.23 | >.05 | 0.73 |
|  |  | | 40 | 100 | 0.09 | >.05 | 0.29 |
|  | UK-06 | | placebo | 23 |  |  |  |
|  |  | | 30 | 22 | 0.33 | >.05 | 0.84 |
| (continued) | | | | | | | |
| Table 1. (continued) |  | |  |  |  |  |  |
| Drug | Trial Number | | Dose (mg/d) | Sample size | Effect size | *p* | *BF* |
|  | UK-09 | | placebo | 21 |  |  |  |
|  |  | | 30 | 20 | 0.38 | >.05 | 0.98 |
|  | UK-12 | | placebo | 10 |  |  |  |
|  |  | | 30 | 19 | 0.64 | >.05 | 1.88 |
| Paroxetine CR | 487 | | placebo | 107 |  |  |  |
|  |  | | 12.5-50 | 103 | 0.37 | .007 | 9.36 |
|  | 449 | | placebo | 110 |  |  |  |
|  |  | | 20-62.5 | 108 | 0.39 | .004 | 15.00 |
|  | 448 | | placebo | 93 |  |  |  |
|  |  | | 20-62.5 | 94 | 0.17 | .254 | 0.51 |
| Sertraline | 104 | | placebo | 141 |  |  |  |
|  |  | | 50-200 | 142 | 0.33 | .006 | 9.70 |
|  | 103 | | placebo | 86 |  |  |  |
|  |  | | 50 | 90 | 0.36 | .018 |  |
|  |  | | 100 | 89 | 0.26 | .084 |  |
|  |  | | 200 | 82 | 0.19 | .21 |  |
|  | 315 | | placebo | 73 |  |  |  |
|  |  | | 50-200 | 75 | 0.12 | .46 | 0.35 |
|  | 101 | | placebo | 23 |  |  |  |
|  |  | | 50 | 22 | 0.28 | .35 | 0.68 |
|  |  | | 100 | 19 | 0.05 | .87 | 0.34 |
|  |  | | 200 | 17 | 0.40 | .21 | 1.02 |
|  |  | | 400 | 12 | 0.16 | .64 | 0.48 |
|  | 310 | | placebo | 30 |  |  |  |
|  |  | | 50 | 31 | 0.16 | >.05 | 0.45 |
|  |  | | 100 | 28 | 0.20 | >.05 | 0.51 |
|  |  | | 200 | 27 | 0.11 | >.05 | 0.38 |
|  |  | | 400 | 30 | 0.33 | >.05 | 0.94 |
| Venlafaxine | 600A-203 | | placebo | 92 |  |  |  |
|  |  | | 75 | 77 | 0.45 | .004 | 16.15 |
|  |  | | 150-225 | 79 | 0.55 | <.001 | 107.44 |
|  |  | | 300-375 | 75 | 0.47 | .003 | 20.76 |
|  | 600A-206 | | placebo | 47 |  |  |  |
|  |  | | 150-375 | 46 | 0.58 | .006 | 13.11 |
|  | 600A-301 | | placebo | 78 |  |  |  |
|  |  | | 75-225 | 64 | 0.61 | <.001 | 135.77 |
|  | 600A-302 | | placebo | 75 |  |  |  |
|  |  | | 75-200 | 65 | 0.45 | .008 | 9.40 |
|  | 600A-303 | | placebo | 79 |  |  |  |
|  |  | | 75-225 | 69 | 0.11 | .493 | 0.33 |
|  | 600A-313 | | placebo | 75 |  |  |  |
|  |  | | 75 | 72 | 0.21 | .193 | 0.69 |
|  |  | | 200 | 77 | 0.24 | .142 | 0.87 |
| Venlafaxine XR | 208 | | placebo | 91 |  |  |  |
|  |  | | 875-150 | 85 | 0.50 | .001 | 53.30 |
|  | 209 | | placebo | 100 |  |  |  |
|  |  | | 75-225 | 91 | 0.50 | <.001 | 82.72 |
| (continued) | | | | | | | |
| Table 1. (continued) | | | | | | | |
| Drug | Trial Number | | Dose (mg/d) | Sample size | Effect size | *p* | *BF* |
|  | 367 | | placebo | 81 |  |  |  |
|  |  | | 75 | 82 | 0.14 | .37 | 0.40 |
|  |  | | 150 | 75 | 0.24 | .14 | 0.87 |
| Vilazodone | 244 | | placebo | 95 |  |  |  |
|  |  | | 20-100 | 86 | -0.10 | .4938 | 0.10 |
|  | 245 | | placebo | 99 |  |  |  |
|  |  | | 40-60 | 97 | 0.04 | .753 | 0.20 |
|  |  | | 80-100 | 93 | -0.22 | .131 | 0.07 |
|  | 246 | | placebo | 129 |  |  |  |
|  |  | | 20 | 123 | 0.10 | .407 | 0.30 |
|  | 247 | | placebo | 111 |  |  |  |
|  |  | | 5-20 | 109 | 0.15 | .273 | 0.45 |
|  | 248 | | placebo | 128 |  |  |  |
|  |  | | 20 | 132 | 0.03 | .802 | 0.17 |
|  | 07 | | placebo | 231 |  |  |  |
|  |  | | 40 | 232 | 0.24 | .009 | 5.66 |
|  | 04 | | placebo | 199 |  |  |  |
|  |  | | 40 | 198 | 0.33 | .001 | 42.24 |
| Vortioxetine | 315 | | placebo | 153 |  |  |  |
|  |  | | 20 | 147 | 0.26 | .023 | 2.99 |
|  | 316 | | placebo | 155 |  |  |  |
|  |  | | 20 | 148 | 0.36 | .002 | 24.88 |
|  | 13267A | | placebo | 158 |  |  |  |
|  |  | | 15 | 149 | 0.58 | NA | 37592.30 |
|  |  | | 20 | 151 | 0.75 | NA | 7.51*10^7^ |
|  | 11492A | | placebo | 105 |  |  |  |
|  |  | | 5 | 108 | 0.58 | NA | 1103.50 |
|  |  | | 10 | 100 | 0.56 | NA | 472.92 |
|  | 305 | | placebo | 139 |  |  |  |
|  |  | | 5 | 139 | 0.47 | NA | 370.11 |
|  |  | | 10 | 139 | 0.56 | NA | 6206.22 |
|  | 12541 | | placebo | 145 |  |  |  |
|  |  | | 5 | 155 | 0.38 | .0011 | 42.48 |
|  | 11984A | | placebo | 145 |  |  |  |
|  |  | | 5 | 155 | 0.17 | .132 | 0.70 |
|  |  | | 10 | 151 | 0.15 | .185 | 0.54 |
|  | 317 | | placebo | 149 |  |  |  |
|  |  | | 10 | 143 | 0.06 | .597 | 0.21 |
|  |  | | 15 | 142 | 0.04 | .745 | 0.17 |
|  | 303 | | placebo | 286 |  |  |  |
|  |  | | 5 | 292 | 0.07 | .407 | 0.21 |
|  | 304 | | placebo | 149 |  |  |  |
|  |  | | 10 | 146 | 0.17 | .138 | 0.68 |
|  |  | | 15 | 153 | 0.06 | .577 | 0.21 |
| (continued) | | | | | | | |
|  | | | | | | | |
| Table 1. (continued) |  | |  |  |  |  |  |
| Drug | Trial Number | | Dose (mg/d) | Sample size | Effect size | *p* | *BF* |
| ***Anxiety*** |  | |  |  |  |  |  |
| *GAD* |  | |  |  |  |  |  |
| Duloxetine | F1J-MC-HMBR | | placebo | 173 |  |  |  |
|  |  | | 60 | 165 | 0.50 | <.001 | 5470.71 |
|  |  | | 120 | 169 | 0.47 | <.001 | 1720.57 |
|  | F1J-MC-HMDT | | placebo | 158 |  |  |  |
|  |  | | 60-120 | 161 | 0.26 | .023 | 2.92 |
|  | F1J-MC-HMDU | | placebo | 158 |  |  |  |
|  |  | | 60-120 | 149 | 0.31 | .007 | 8.25 |
| Escitalopram | SCT-MD-05 | | placebo | 128 |  |  |  |
|  |  | | 10-20 | 124 | 0.25 | .044 | 1.86 |
|  | SCT-MD-06 | | placebo | 138 |  |  |  |
|  |  | | 10-20 | 143 | 0.26 | .032 | 2.32 |
|  | SCT-MD-07 | | placebo | 153 |  |  |  |
|  |  | | 10-20 | 154 | 0.52 | <.001 | 4706.78 |
| Paroxetine | 641 | | placebo | 180 |  |  |  |
|  |  | | 20 | 188 | 0.39 | <.01 | 165.57 |
|  |  | | 40 | 197 | 0.34 | <.01 | 43.96 |
|  | 642 | | placebo | 163 |  |  |  |
|  |  | | 20-50 | 161 | 0.30 | .0077 | 7.44 |
|  | 637 | | placebo | 183 |  |  |  |
|  |  | | 20-50 | 181 | 0.11 | .2808 | 0.35 |
| Venlafaxine XR | 210 | | placebo | 96 |  |  |  |
|  |  | | 75 | 86 | 0.19 | .20 | 0.62 |
|  |  | | 150 | 81 | 0.27 | .07 | 1.46 |
|  |  | | 225 | 86 | 0.32 | .03 | 2.88 |
|  | 214 | | placebo | 98 |  |  |  |
|  |  | | 75 | 87 | 0.38 | .01 | 7.19 |
|  |  | | 150 | 87 | 0.24 | .10 | 1.07 |
| *OCD*  Fluoxetine | HCEP study 1 | | placebo | 47 |  |  |  |
|  |  | | 20 | 47 | 0.70 | 0 | 59.52 |
|  |  | | 40 | 45 | 0.55 | .01 | 9.42 |
|  |  | | 60 | 47 | 0.54 | .01 | 8.59 |
|  | HCEP study 2 | | placebo | 41 |  |  |  |
|  |  | | 20 | 39 | 0.51 | .03 | 4.25 |
|  |  | | 40 | 41 | 0.95 | <.001 | 1033.54 |
|  |  | | 60 | 42 | 1.15 | <.001 | 27256.92 |
|  | E079 | | placebo | 56 |  |  |  |
|  |  | | 20 | 52 | 0.26 | .18 | 0.83 |
|  |  | | 40 | 52 | 0.12 | .55 | 0.34 |
|  |  | | 60 | 54 | 0.36 | .06 | 1.94 |
| Fluvoxamine | 5529 | | placebo | 80 |  |  |  |
|  |  | | 100-300 | 79 | 0.60 | 0 | 226.36 |
|  | 5534 | | placebo | 77 |  |  |  |
|  |  | | 100-300 | 78 | 0.40 | .01 | 6.08 |
| (continued) | | | | | | | |
| Table 1. (continued) | | | | | | | |
| Drug | Trial Number | | Dose (mg/d) | Sample size | Effect size | *p* | *BF* |
| Fluvoxamine CR | 3103 | | placebo | 119 |  |  |  |
|  |  | | 100-300 | 113 | 0.44 | 0 | 49.81 |
| Paroxetine | 116 | | placebo | 88 |  |  |  |
|  |  | | 40 | 83 | 0.39 | .01 | 6.31 |
|  |  | | 60 | 83 | 0.48 | 0 | 29.29 |
|  | 118 | | placebo | 75 |  |  |  |
|  |  | | 20-60 | 79 | 0.14 | .40 | 0.38 |
|  | 136 | | placebo | 99 |  |  |  |
|  |  | | 20-60 | 198 | 0.30 | .02 | 4.27 |
| Sertraline | 237/248 | | placebo | 44 |  |  |  |
|  |  | | 50-200 | 43 | 0.41 | .056 | 2.20 |
|  | 549 | | placebo | 79 |  |  |  |
|  |  | | 50-200 | 85 | 0.41 | .01 | 7.45 |
|  | 495 | | placebo | 87 |  |  |  |
|  |  | | 50-200 | 83 | 0.01 | >.10 | 0.17 |
|  | 371/372 | | placebo | 84 |  |  |  |
|  |  | | 50 | 79 | 0.43 | .007 | 10.10 |
|  |  | | 100 | 81 | 0.18 | 259 | 0.52 |
|  |  | | 200 | 80 | 0.45 | .004 | 16.26 |
| *PD* |  | |  |  |  |  |  |
| Fluoxetine | HCJC | | placebo | 90 |  |  |  |
|  |  | | 20-60 | 90 | 0.34 | .018 | 4.42 |
|  | HCJB | | placebo | 104 |  |  |  |
|  |  | | 20-60 | 107 | 0.40 | .008 | 8.34 |
| Paroxetine | 120 | | placebo | 69 |  |  |  |
|  |  | | 40 | 72 | 0.76 | .025 | 4.21*10^27^ |
|  | 108 | | placebo | 60 |  |  |  |
|  |  | | 20-60 | 60 | 0.59 | <.05 | 263636.35 |
|  | 187 | | placebo | 123 |  |  |  |
|  |  | | 20-60 | 123 | 0.41 | <.05 | 7.21*10^11^ |
|  | 223 | | placebo | 68 |  |  |  |
|  |  | | 10-60 | 77 | -0.09 | >.10 | 0.22 |
| Paroxetine CR | 494 | | placebo | 129 |  |  |  |
|  |  | | 25-75 | 122 | 0.43 | .004 | 14.37 |
|  | 495 | | placebo | 136 |  |  |  |
|  |  | | 25-75 | 123 | 0.12 | .217 | 0.50 |
|  | 497 | | placebo | 130 |  |  |  |
|  |  | | 25-75 | 132 | 0.13 | .127 | 0.77 |
| Sertraline | 629 | | placebo | 87 |  |  |  |
|  |  | | 50-200 | 79 | 0.49 | .002 | 29.51 |
|  | 630 | | placebo | 88 |  |  |  |
|  |  | | 50-200 | 88 | 0.23 | .12 | 0.94 |
|  | 529 | | placebo | 44 |  |  |  |
|  |  | | 50 | 42 | 0.23 | .283 | 0.63 |
|  |  | | 100 | 41 | 0.41 | .062 | 2.05 |
|  |  | | 200 | 44 | 0.11 | .619 | 0.34 |
| (continued) | | | | | | | |
| Table 1. (continued) | | | | | | | |
| Drug | Trial Number | | Dose (mg/d) | Sample size | Effect size | *p* | *BF* |
|  | 514 | | placebo | 38 |  |  |  |
|  |  | | 50 | 38 | -0.15 | .523 | 0.16 |
|  |  | | 100 | 38 | 0.40 | .081 | 1.72 |
|  |  | | 200 | 36 | 0.28 | .224 | 0.80 |
| Venlafaxine XR | 398 | | placebo | 154 |  |  |  |
|  |  | | 75 | 157 | 0.45 | <.001 | 1.92*10^25^ |
|  |  | | 150 | 158 | 0.61 | <.001 | 4.56*10^64^ |
|  | 399 | | placebo | 157 |  |  |  |
|  |  | | 75 | 156 | 0.40 | <.001 | 1.32*10^17^ |
|  |  | | 225 | 160 | 0.54 | <.001 | 1.03*10^47^ |
|  | 353 | | placebo | 155 |  |  |  |
|  |  | | 75-225 | 155 | 0.23 | .056 | 1.40 |
|  | 391 | | placebo | 168 |  |  |  |
|  |  | | 75-225 | 160 | 0.06 | .622 | 0.19 |
| *PTSD*  Paroxetine | 651 | | placebo | 167 |  |  |  |
|  |  | | 20 | 166 | 0.56 | <.001 | 57863.28 |
|  |  | | 40 | 156 | 0.48 | <.001 | 1569.42 |
|  | 648 | | placebo | 133 |  |  |  |
|  |  | | 20-50 | 136 | 0.48 | .0001 | 381.03 |
|  | 627 | | placebo | 159 |  |  |  |
|  |  | | 20-50 | 154 | 0.24 | .0363 | 2.00 |
| Sertraline | 641 | | placebo | 82 |  |  |  |
|  |  | | 50-200 | 84 | -0.08 | .587 | 0.12 |
|  | 682 | | placebo | 94 |  |  |  |
|  |  | | 50-200 | 94 | -0.02 | .896 | 0.14 |
|  | 640 | | placebo | 104 |  |  |  |
|  |  | | 50-200 | 98 | 0.29 | .043 | 2.06 |
|  | 671 | | placebo | 90 |  |  |  |
|  |  | | 50-200 | 93 | 0.36 | .016 | 4.85 |
| *SAD* |  | |  |  |  |  |  |
| Fluvoxamine CR | 3107 | | placebo | 125 |  |  |  |
|  |  | | 100-300 | 110 | 0.56 | <.0001 | 1424.59 |
|  | 3108 | | placebo | 148 |  |  |  |
|  |  | | 100-300 | 126 | 0.28 | .023 | 3.11 |
| Paroxetine | 502 | | placebo | 145 |  |  |  |
|  |  | | 20-50 | 136 | 0.42 | <.001 | 87.95 |
|  | 382 | | placebo | 92 |  |  |  |
|  |  | | 20-50 | 90 | 0.63 | <.001 | 1202.16 |
|  | 454 | | placebo | 92 |  |  |  |
|  |  | | 20 | 89 | 0.50 | .001 | 53.00 |
|  |  | | 40 | 88 | 0.31 | .039 | 2.33 |
|  |  | | 60 | 91 | 0.34 | .024 | 3.46 |
| Paroxetine CR | 790 | | placebo | 184 |  |  |  |
|  |  | | 12.5-37.5 | 185 | 0.55 | <.001 | 131400.43 |
| Sertraline | R-0601 | | placebo | 196 |  |  |  |
|  |  | | 50-200 | 205 | 0.33 | .001 | 42.11 |
| (continued) | | | | | | | |
| Table 1. (continued) | | | | | | | |
| Drug | Trial Number | | Dose (mg/d) | Sample size | Effect size | *p* | *BF* |
|  | STL-NY-94-004 | | placebo | 69 |  |  |  |
|  |  | | 50-200 | 134 | 0.49 | .001 | 52.73 |
|  | 95-003 | | placebo | 196 |  |  |  |
|  |  | | 50-150 | 191 | 0.09 | >.05 | 0.27 |
| Venlafaxine ER | 387 | | placebo | 138 |  |  |  |
|  |  | | 75-225 | 133 | 0.40 | .001 | 47.68 |
|  | 393 | | placebo | 135 |  |  |  |
|  |  | | 75-225 | 126 | 0.37 | .003 | 18.26 |
| ADHD |  | |  |  |  |  |  |
| Adderall XR | 301 | | placebo | 203 |  |  |  |
|  |  | | 10 | 128 | 0.86 | <.001 | 4.54*10^10^ |
|  |  | | 20 | 112 | 0.94 | <.001 | 4.73*10^11^ |
|  |  | | 30 | 120 | 1.00 | <.001 | 6.54*10^13^ |
|  | 303 | | placebo | 60 |  |  |  |
|  |  | | 20 | 64 | 0.60 | .001 | 57.17 |
|  |  | | 40 | 64 | 0.67 | <.001 | 183.81 |
|  |  | | 60 | 60 | 0.66 | <.001 | 119.78 |
|  | 314 | | placebo | 52 |  |  |  |
|  |  | | 10 | 54 | 0.56 | .0043 | 16.91 |
|  |  | | 20 | 53 | 1.03 | <.0001 | 42190.12 |
|  |  | | 30 | 58 | 0.88 | <.0001 | 3324.74 |
|  |  | | 40 | 61 | 0.63 | <.0001 | 56.90 |
|  | 201*^a^* | | 10 | 49 | 0.50 | <.001 | 334.75 |
|  |  | | 20 | 49 | 0.73 | <.0001 | 2.1*10^5^ |
|  |  | | 30 | 49 | 0.64 | <.0001 | 13479.43 |
| Adhansia XR | 063-009 | | placebo | 71 |  |  |  |
|  |  | | 25 | 71 | 0.19 | .2562 | 0.56 |
|  |  | | 45 | 68 | 0.48 | .0052 | 13.55 |
|  |  | | 70 | 72 | 0.46 | .0069 | 10.59 |
|  |  | | 85 | 70 | 0.39 | .0226 | 3.95 |
|  | 063-010 | | placebo | 77 |  |  |  |
|  |  | | 25 | 75 | 0.17 | .3016 | 0.48 |
|  |  | | 45 | 73 | 0.62 | .0002 | 228.45 |
|  |  | | 70 | 71 | 0.20 | .2287 | 0.61 |
|  |  | | 100 | 72 | 0.67 | <.0001 | 605.54 |
|  | 063-015 | | placebo | 73 |  |  |  |
|  |  | | 25-85 | 74 | 0.17 | <.0001 | 0.47 |
|  | 063-008*^a^* | | 25-100 | 45 | 0.17 | .0064 | 14.78 |
| Aptensio XR | RP-BP-EF002 | | placebo | 46 |  |  |  |
|  |  | | 10 | 48 | 0.26 | .2083 | 0.78 |
|  |  | | 15 | 40 | 0.38 | .0769 | 1.73 |
|  |  | | 20 | 44 | 0.52 | .0145 | 6.41 |
|  |  | | 40 | 43 | 0.71 | .0011 | 55.22 |
|  | RP-BP-EF001 *^a^* | | variable dose | 20 | 0.76 | .0001 | 1891.35 |
| Concerta | C-98-005 | | placebo | 90 |  |  |  |
|  |  | | 18-54 | 95 | 0.88 | <.001 | 2150044 |
| (continued) | | | | | | | |
|  | | | | | | | |
| Table 1. (continued) | | | | | | | |
| Drug | Trial Number | | Dose (mg/d) | Sample size | Effect size | *p* | *BF* |
|  | 3002 | | placebo | 95 |  |  |  |
|  |  | | 18 | 99 | 0.29 | .0146 | 2.18 |
|  |  | | 36 | 101 | 0.39 | .0131 | 9.97 |
|  |  | | 72 | 99 | 0.58 | <.0001 | 490.39 |
|  | 02-159 | | placebo | 116 |  |  |  |
|  |  | | 36-108 | 110 | 0.34 | .012 | 5.77 |
|  | C-98-003 | | 18-54 | 61 | 1.37 | <.001 | 3.47*10^13^ |
|  | C-97-025 | | 18-54 | 67 | 1.48 | <.001 | 1.43*10^19^ |
| Cotempla XR | NT0102.1004 | | placebo | 39 |  |  |  |
|  |  | | 20-60 | 43 | 1.69 | <.0001 | 4.51*10^8^ |
| Daytrana | 302 | | placebo | 85 |  |  |  |
|  |  | | individualized dose | 96 | 0.97 | <.0001 | 3.34*10^7^ |
|  | 10 | | placebo | 101 |  |  |  |
|  |  | | 6.25-25.0 cm^2^ | 98 | 0.04 | .7927 | 0.19 |
|  | 18 | | placebo | 102 |  |  |  |
|  |  | | 6.25-50 cm^2^ | 101 | 1.16 | <.0001 | 5.96*10^11^ |
|  | 201 | | individualized dose | 79 | 0.93 | <.0001 | 5.85*10^11^ |
| Dyanavel XR | NTRI102-ADD-001 | | placebo | 48 |  |  |  |
|  |  | | 2.5-20 | 51 | 1.89 | <.0001 | 5.27*10^12^ |
| Evekeo ODT | AR11.001^a^ | | 10-40 | 97 | 0.77 | <.0001 | 2.53*10^8^ |
| Focalin | 97-M-02 | | placebo | 39 |  |  |  |
|  |  | | 5-20 | 42 | 0.68 | <.0001 | 24.06 |
|  | 97-M-03 | | placebo | 39 |  |  |  |
|  |  | | 5-20 | 35 | 0.79 | .001 | 60.99 |
| Focalin XR | 2301 | | placebo | 45 |  |  |  |
|  |  | | 5-30 | 52 | 0.68 | <.001 | 55.90 |
|  | 2302 | | placebo | 53 |  |  |  |
|  |  | | 20 | 57 | 0.53 | .006 | 12.69 |
|  |  | | 30 | 54 | 0.49 | .012 | 7.18 |
|  |  | | 40 | 54 | 0.72 | <.001 | 186.18 |
| Intuniv | 301 | | placebo | 78 |  |  |  |
|  |  | | 2 | 84 | 0.55 | .0006 | 85.14 |
|  |  | | 3 | 82 | 0.56 | .0005 | 100.24 |
|  |  | | 4 | 81 | 0.67 | <.0001 | 938.52 |
|  | 304 | | placebo | 63 |  |  |  |
|  |  | | 1 | 57 | 0.53 | .0041 | 17.16 |
|  |  | | 2 | 63 | 0.43 | .0176 | 5.02 |
|  |  | | 3 | 60 | 0.58 | .0016 | 38.13 |
|  |  | | 4 | 63 | 0.62 | .0006 | 88.97 |
| Jornay PM | HLD200-107 | | placebo | 71  82 | 0.42 | .01 | 7.61 |
|  |  |  |  |  |  |  |  |
|  | HLD200-108 | placebo | | 80  81 | 0.49 | .002 | 29.71 |
|  |  |  | |  |  |  |  |
| (continued) | | | | | | | |
| Table 1. (continued) | | | | | | | |
| Drug | Trial Number | | Dose (mg/d) | Sample size | Effect size | *p* | *BF* |
| Kapvay | 301 | | placebo | 76 |  |  |  |
|  |  | | 0.2 | 74 | 0.77 | <.0001 | 5787.14 |
|  |  | | 0.4 | 78 | 0.78 | <.0001 | 9724.42 |
|  | 302 | | placebo | 95 |  |  |  |
|  |  | | 0.1-0.4 | 102 | 0.37 | .0091 | 7.64 |
| Metadate CD | 1001-04 | | placebo | 159 |  |  |  |
|  |  | | 20-60 | 155 | 1.04 | <.001 | 2.35*10^15^ |
| Mydayis | SHP465-305 | | placebo | 129 |  |  |  |
|  |  | | 12.5-25 | 128 | 0.78 | <.001 | 1.33*10^7^ |
|  | SHP465-306 | | placebo | 86 |  |  |  |
|  |  | | 12.5 | 89 | 0.66 | <.001 | 1742.9 |
|  |  | | 37.5 | 88 | 1.08 | <.001 | 6.21*10^8^ |
| QuilliChew ER | B7491005 | | placebo | 43 |  |  |  |
|  |  | | 20-60 | 42 | 0.76 | <.001 | 80.80 |
| Quillivant XR *^a^* | NWP06-ADD-100 | | 20-60 | 39 | 1.74 | <.0001 | 3.70*10^14^ |
| Ritalin LA | 07 | | placebo | 70 |  |  |  |
|  |  | | individual dose | 62 | 1.02 | <.0001 | 6.01*10^5^ |
| Strattera | HFBD | | placebo | 61 |  |  |  |
|  |  | | 10-180 | 64 | 0.72 | .0001 | 433.27 |
|  | HFBK | | placebo | 60 |  |  |  |
|  |  | | 10-180 | 63 | 0.64 | .0005 | 104.73 |
|  | LYAC | | placebo | 83 |  |  |  |
|  |  | | 1.2 mg/kg/day | 84 | 0.62 | <.001 | 457.64 |
|  |  | | 1.8 mg/kg/day | 82 | 0.60 | <.001 | 268.76 |
|  | LYAT | | placebo | 83 |  |  |  |
|  |  | | 0.5-1.5 mg/kg/day | 84 | 0.62 | <.001 | 513.93 |
|  | LYAA | | placebo | 134 |  |  |  |
|  |  | | 60-120 | 133 | 0.35 | .004 | 14.08 |
|  | LYAO | | placebo | 124 |  |  |  |
|  |  | | 60-120 | 124 | 0.43 | <.001 | 53.14 |
| Vyvanse | 301 | | placebo | 72 |  |  |  |
|  |  | | 30 | 69 | 0.99 | <.0001 | 9.16*10^5^ |
|  |  | | 50 | 71 | 1.10 | <.0001 | 2.74*10^7^ |
|  |  | | 70 | 73 | 1.30 | <.0001 | 1.72*10^10^ |
|  | 201 | | individualized dose | 50 | 0.92 | <.0001 | 6.84*10^11^ |

Note: For ADHD medication we used commercial Names instead of the active agent a = cross-over trial; trials considered as "failed" by the FDA were considered "negative"

## Visual presentation of individual Bayes Factors

**
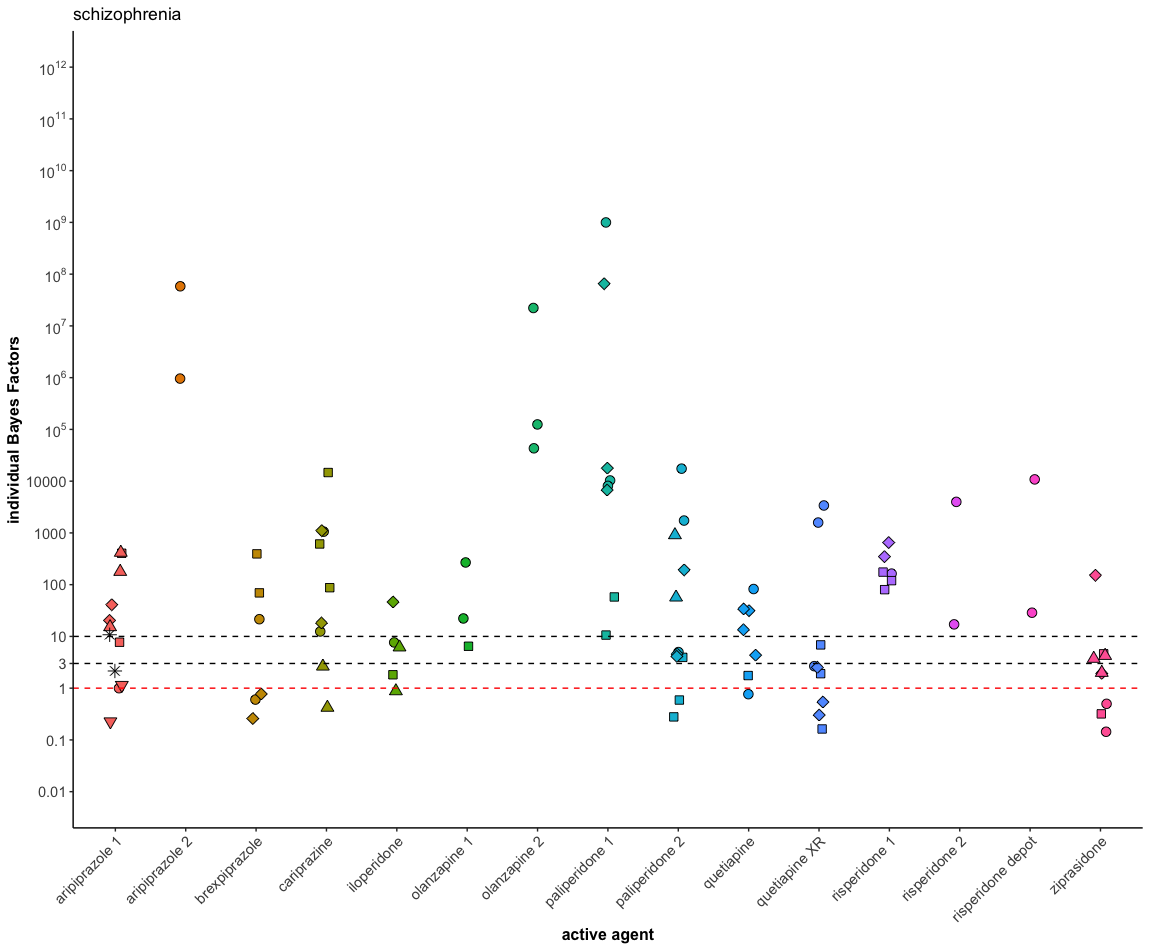
**Figure 1 through 4 visually present the individual Bayes Factors.

**Figure S1**

*Individual trial Bayes Factors for all effect included for antipsychotics. Colors represent different drugs. Shapes represent different trials. We highlight three thresholds: BF=1, BF=3, and BF=10. Numbers are used to differentiate drugs with the same non-proprietary name.*

**
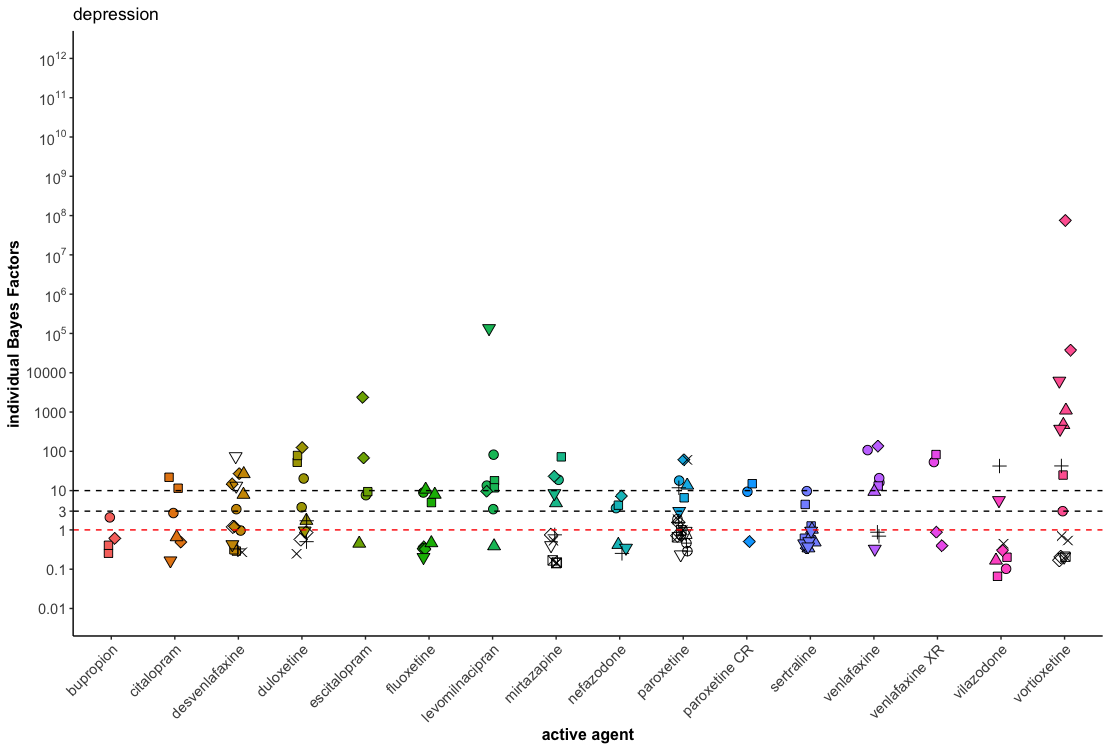
Figure S2**

*Individual trial Bayes Factors for all effect included for antidepressants approved for depression. Colors represent different drugs. Shapes represent different trials. We highlight three thresholds: BF=1, BF=3, and BF=10.*

**
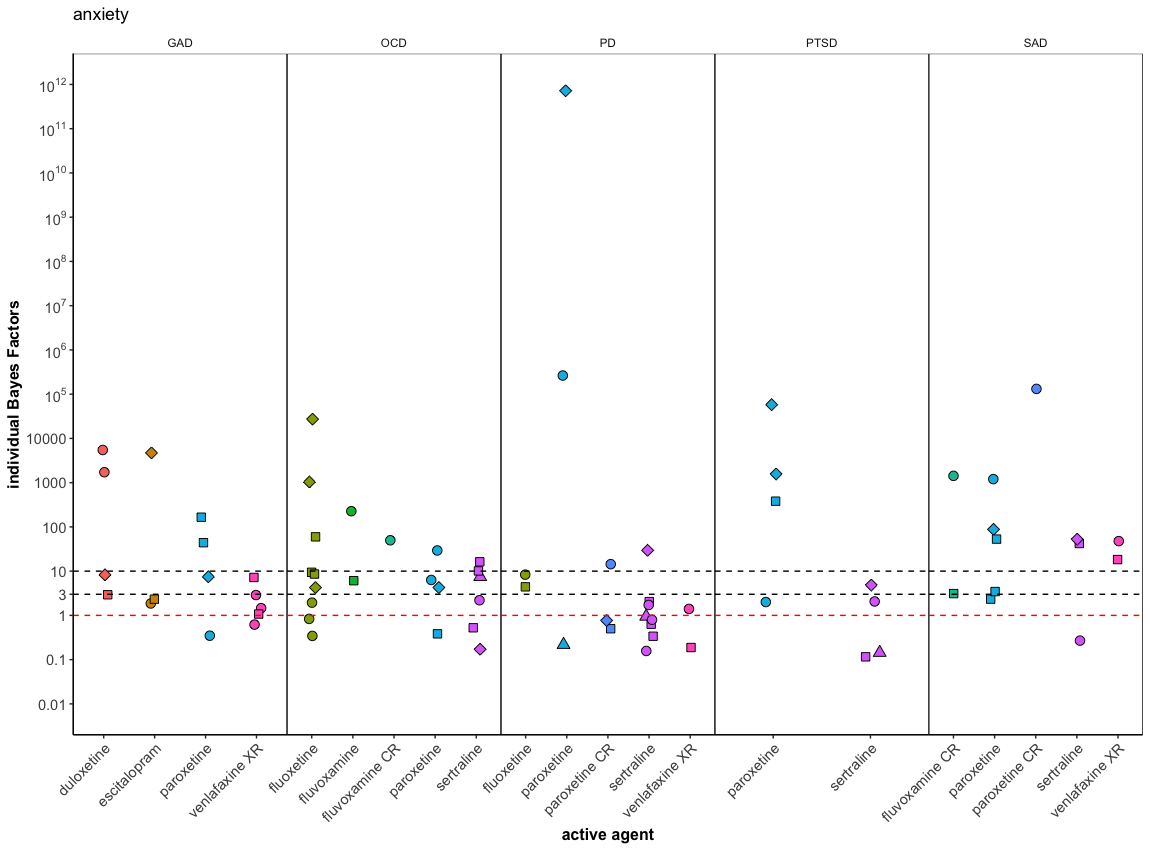
Figure S3.**

*Individual trial Bayes Factors for all effect included for antidepressants approved for anxiety disorders. Colors represent different drugs. Shapes represent different trials. We highlight three thresholds: BF=1, BF=3, and BF=10.*

**
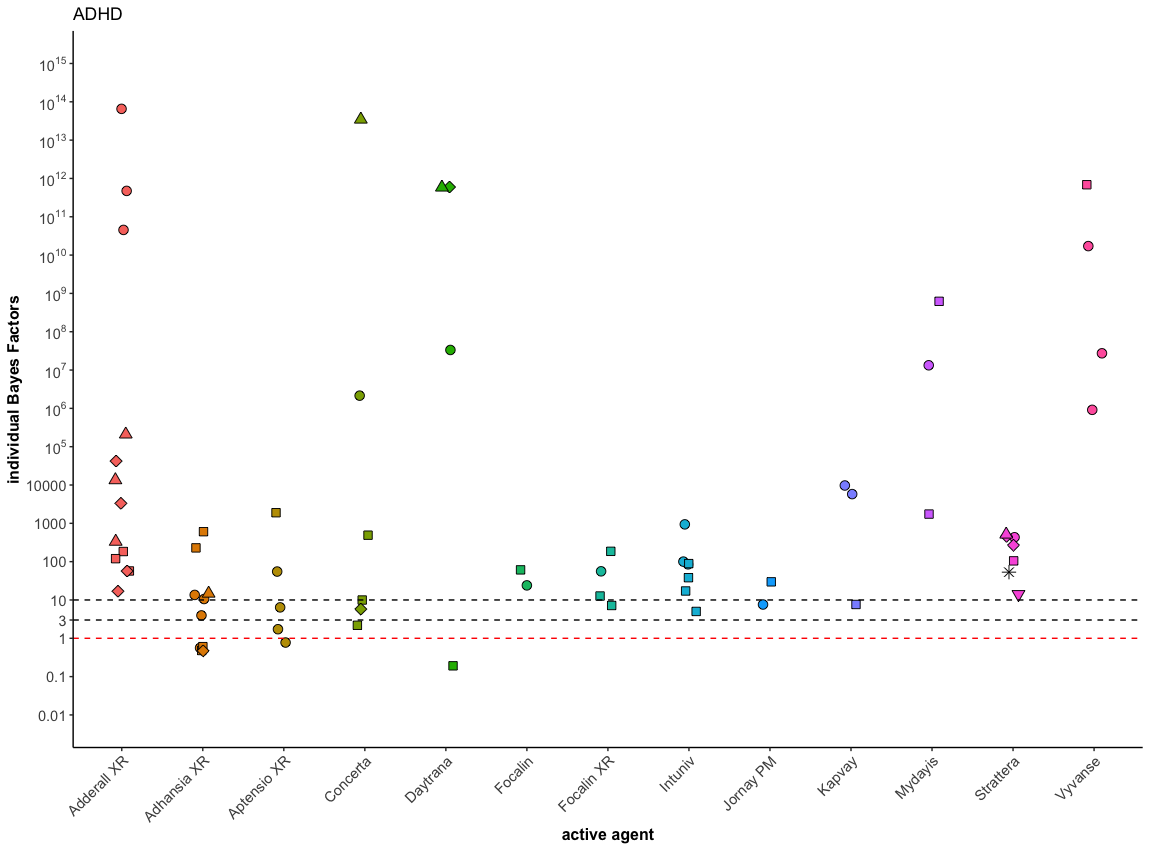
**

**Figure S4**

*Individual trial Bayes Factors for all effect included for ADHD medication. Colors represent different drugs. Shapes represent different trials. We highlight three thresholds: BF=1, BF=3, and BF=10.*

## Meta-analytic Bayes Factors

Table S2 summarizes the results from all meta-analyses performed.

| Table S2  *Summary of all main and sensitivity analyses. Colours highlight BFs below 30, 10, or 3.* | | | | | | | | | | | | | |
| --- | --- | --- | --- | --- | --- | --- | --- | --- | --- | --- | --- | --- | --- |
| Drug | Nr. trials | | N  [min, max] | *ES*_pooled_ | *BF*_BMA_^*^ | *BF*_fixed_ | *BF*_random_ | *BF*_optimistic_ | *BF*_pessimistic_ | *BF*_fixed first_ | | *BF*_imputed_ | *BF*_cross-over_ |
| ***Schizophrenia*** |  | |  |  |  |  |  |  |  |  | |  |  |
| Aripiprazole (Abilify) | 6 | | [68, 410] | 0.41 | 8.0*10^5^ | 3.1*10^17^ | 2.3*10^5^ | 3.4*10_5_ | 8.1*10^5^ | 1245.2 | |  |  |
| Aripiprazole (Aristada) | 1 | | 589 | 0.60 | 74.0 | 2.2*10^14^ | 28.4 | 39.7 | 52.6 |  | |  |  |
| Brexpiprazole | 3 | | [275, 540] | 0.28 | 164.2 | 3.5*10^6^ | 60.98 | 62.3 | 244.2 | 13.8 | |  |  |
| Cariprazine | 4 | | [377, 573] | 0.40 | 55187.2 | 2.2*10^20^ | 18931.6 | 20267.6 | 50026.2 | 124.1 | | 47851.7 |  |
| Iloperidone | 4 | | [232, 523] | 0.27 | 328.9 | 2.8*10^5^ | 100.2 | 124.6 | 492.5 | 145.6 | |  |  |
| Olanzapine (Zyprexa) | 2 | | [98, 189] | 0.56 | 133.1 | 2.4*10^5^ | 51.8 | 68.8 | 103.8 | 34.8 | | 24.5 |  |
| Olanzapine (Zyprexa relprevv) | 1 | | 402 | 0.79 | 498.9 | 1.2*10^18^ | 189.0 | 327.9 | 281.3 |  | |  |  |
| Paliperidone (Invega) | 3 | | [236, 500] | 0.63 | 59668.9 | 4.5*10^37^ | 27796.5 | 32672.3 | 40023.3 | 72.5 | |  |  |
| Paliperidone (Invega sustenna) | 4 | | [197, 636] | 0.39 | 36118.4 | 8.5*10^18^ | 14431.8 | 15147.9 | 38754.3 | 56.6 | |  |  |
| Quetiapine | 3 | | [106, 280] | 0.45 | 3642.7 | 3.6*10^8^ | 1348.0 | 1634.0 | 3428.4 | 54.2 | |  |  |
| Quetiapine XR | 3 | | [333, 454] | 0.30 | 168.7 | 1.8*10^8^ | 102.0 | 240.3 | 816.3 | 7.0 | |  |  |
| Risperidone depot | 1 | | 283 | 0.55 | 29.6 | 4.8*10^5^ | 13.2 | 15.2 | 23.4 |  | |  |  |
| Risperidone (Risperdal) | 3 | | [104, 341] | 0.63 | 23594.5 | 1.7*10^16^ | 7865.1 | 11837.4 | 14651.1 | 211.3 | | 63.9 |  |
| Risperidone (Perseris) | 1 | | 259 | 0.54 | 26.7 | 97253.4 | 12.1 | 13.6 | 21.7 |  | |  |  |
| Ziprasidone | 4 | | [131, 324]] | 0.30 | 365.4 | 1.9*10^5^ | 136.5 | 141.8 | 505.3 | 22.7 | |  |  |
| ***Depression*** |  | |  |  |  |  |  |  |  |  | |  |  |
| Bupropion | 3 | | [230, 338] | 0.16 | 2.7 | 3.6 | 1.8 | 1.0 | 5.9 | 2.4 | |  |  |
| Citalopram | 5 | | [125, 354] | 0.26 | 54.4 | 1324.3 | 21.4 | 20.4 | 86.3 | 16.5 | |  |  |
| Desvenlafaxine | 9 | | [213, 483] | 0.26 | 3.3*10^5^ | 10.*10^11^ | 91027.5 | 1.2*10^5^ | 5.2*10^5^ | 1131.1 | |  |  |
| Duloxetine | 8 | | [113,295] | 0.31 | 42376.8 | 3.3*10^10^ | 12550.5 | 15821.3 | 54004.2 | 911.5 | | 4177.4 |  |
| Escitalopram | 4 | | [249, 377] | 0.35 | 84.4 | 1.4*10^8^ | 41.5 | 55.4 | 172.4 | 37.9 | | 69.4 |  |
| Fluoxetine | 5 | | [42,365] | 0.25 | 104.1 | 1586.8 | 39.4 | 38.6 | 172.0 | 12.8 | |  |  |
| (continued) | | | | | | | | | | | | | |
| Table S2 (continued) | | | | | | | | | | | | | |
| Drug | Nr. trials | | N  [min, max] | *ES*_pooled_ | *BF*_BMA_ | *BF*_fixed_ | *BF*_random_ | *BF*_optimistic_ | *BF*_pessimistic_ | *BF*_fixed first_ | | *BF*_imputed_ | *BF*_cross-over_ |
| Levomilnacipran | 5 | | [355, 704] | 0.31 | 9546.7 | 4.7*10^13^ | 3038.6 | 3706.9 | 12819.8 | 246.2 | |  |  |
| Mirtazapine | 10 | | [80, 125] | 0.29 | 17.4 | 11210.0 | 11.7 | 6.7 | 25.5 | 27.1 | |  |  |
| Nefazodone | 6 | | [88,177] | 0.25 | 32.4 | 117.3 | 14.7 | 12.1 | 52.7 | 32.4 | |  |  |
| Paroxetine | 16 | | [25, 354] | 0.35 | 10267.8 | 9.4*10^7^ | 5728.2 | 66631.8 | 1.7*10^5^ | 48335.1 | | 12752.4 |  |
| Paroxetine CR | 3 | | [187, 218] | 0.31 | 22.0 | 311.3 | 8.9 | 8.7 | 30.5 | 22.0 | |  |  |
| Sertraline | 5 | | [93, 347] | 0.24 | 712.3 | 3101.3 | 246.9 | 294.4 | 1307.7 | 82.1 | | 215.2 |  |
| Venlafaxine | 6 | | [93, 323] | 0.40 | 7338.9 | 1.4*10^10^ | 2740.6 | 3106.0 | 7681.4 | 222.9 | | 664.4 |  |
| Venlafaxine XR | 3 | | [176, 238] | 0.34 | 35.6 | 4899.0 | 15.5 | 14.4 | 43.9 | 17.8 | | 8.2 |  |
| Vilazodone | 6 | | [181, 463] | 0.11 | 0.83 | 4.5 | **0.5** | 0.3 | 2.1 | 1.0 | |  |  |
| Vortioxetine | 10 | | [300, 606] | 0.31 | 5054.6 | 1.0*10^24^ | **5054.3** | 1975.9 | 6746.3 | 64.0 | |  |  |
| ***Anxiety*** |  | |  |  |  |  |  |  |  |  | |  |  |
| *GAD* |  | |  |  |  |  |  |  |  |  | |  |  |
| Duloxetine | 3 | | [307, 507] | 0.38 | 177.1 | 2.5*109 | 67.6 | 74.2 | 194.4 | 34.7 | |  |  |
| Escitalopram | 3 | | [252, 307] | 0.34 | 22.9 | 44155.5 | 10.3 | 9.3 | 28.5 | 22.9 | |  |  |
| Paroxetine | 3 | | [324, 565] | 0.28 | 51.6 | 1.7*10^5^ | 20.3 | 19.7 | 75.9 | 13.1 | |  |  |
| Venlafaxine XR | 2 | | [272, 349] | 0.28 | 128.2 | 993.7 | 44.8 | 48.8 | 192.1 | 14.0 | |  |  |
| *OCD* |  | |  |  |  |  |  |  |  |  | |  |  |
| Fluoxetine | 3 | | [163, 214] | 0.53 | 691.8 | 2.2*10^11^ | 462.7 | 340.6 | 556.6 | 7.8 | |  |  |
| Fluvoxamine | 2 | | [155, 159] | 0.48 | 23.7 | 2459.1 | 10.2 | 11.2 | 22.0 | 23.7 | |  |  |
| Fluvoxamine CR | 1^a^ | | 232 | 0.44 | 49.8 |  |  |  |  |  | |  |  |
| Paroxetine | 3 | | [154, 297] | 0.32 | 56.3 | 2588.5 | 21.3 | 22.3 | 75.1 | 18.5 | |  |  |
| Sertraline | 4 | | [87, 324] | 0.30 | 71.8 | 3356.7 | 31.0 | 27.8 | 101.8 | 17.5 | | 277.6 |  |
| *PD* |  | |  |  |  |  |  |  |  |  | |  |  |
| Fluoxetine | 2 | | [180, 211] | 0.36 | 15.5 | 154.1 | 6.5 | 6.4 | 18.9 | 15.5 | |  |  |
| Paroxetine | 4 | | [180, 369] | 0.38 | 4.4 | 50000 | 3.8 | 1.9 | 5.2 | 4.4 | | 4.4 |  |
| Paroxetine CR | 3 | | [251, 262] | 0.22 | 4.4 | 18.1 | 2.3 | 1.6 | 8.0 | 4.4 | |  |  |
| Sertraline | 4 | | [150, 176] | 0.27 | 52.1 | 254.1 | 22.6 | 19.7 | 81.5 | 28.2 | |  |  |
| Venlafaxine XR | 4 | | [310, 473] | 0.37 | 48.6 | 1.4*10^13^ | **41.2** | 20.2 | 55.9 | 6.2 | |  |  |
| *PTSD* |  | |  |  |  |  |  |  |  |  | |  |  |
| Paroxetine | 3 | | [269, 489] | 0.43 | 173.8 | 4.5*10^11^ | 74.7 | 76.9 | 168.0 | 36.9 | |  |  |
| Sertraline | 4 | | [166, 202] | 0.41 | 0.7 | 1.0 | 0.6 | 0.3 | 1.7 | 0.7 | |  |  |
| (continued) | | | | | | | | | | | | | |
| Table S2 (continued) |  | |  |  |  |  |  |  |  |  | |  |  |
| Drug | Nr. trials | | N  [min, max] | *ES*_pooled_ | *BF*_BMA_ | *BF*_fixed_ | *BF*_random_ | *BF*_optimistic_ | *BF*_pessimistic_ | *BF*_fixed first_ | | *BF*_imputed_ | *BF*_cross-over_ |
| *SAD* |  | |  |  |  |  |  |  |  |  | |  |  |
| Fluvoxamine CR | 2 | | [235, 274] | 0.39 | 11.4 | 4099.7 | 5.6 | 4.9 | 12.7 | 11.4 | |  |  |
| Paroxetine | 3 | | [182, 360] | 0.43 | 831.8 | 1.3*10^9^ | 290.5 | 366.3 | 812.4 | 95.9 | |  |  |
| Paroxetine CR | 1^a^ | | 369 | 0.55 | 1.3*10^5^ |  |  |  |  |  | |  |  |
| Sertraline | 3 | | [203, 401] | 0.19 | 7.3 | 4.3 | 7.3 | 2.8 | 11.5 | 7.3 | | 19.7 |  |
| Venlafaxine XR | 2 | | [261, 271] | 0.37 | 23.4 | 2632.0 | 9.0 | 9.8 | 27.1 | 23.4 | |  |  |
| **ADHD** |  | |  |  |  |  |  |  |  |  | |  |  |
| Adderall XR | 4 | | [196, 563] | 0.75 | 1.2*10^8^ | 9.3*10^66^ | 5.9*10^7^ | 5.6*10^7^ | 5.1*10^7^ | 373.5 | | 7.5*10^6^ | 7.5*10^6^ |
| Adhansia XR | 4 | | [90, 368] | 0.30 | 643.6 | 6.1*10^8^ | 448.1 | 249.2 | 896.8 | 21.2 | |  | 1331.8 |
| Aptension XR | 2 | | [40, 221] | 0.50 | 305.2 | 2.3*10^5^ | 124.5 | 146.7 | 260.1 | 25.2 | |  | 82.7 |
| Concerta | 5 | | [122, 394] | 0.69 | 39.0 | 5.3*10^26^ | **39.0** | 23.5 | 26.4 | 18.6 | |  | 89.5 |
| Contempla XR | 1 | | 82 | 1.96 | 4.5*10^8^ |  |  |  |  |  | |  |  |
| Daytrana | 4 | | [158, 203] | 0.67 | 8.3 | 8.9*10^24^ | **8.3** | 5.0 | 6.2 | 8.2 | |  | 3.2 |
| Daynavel XR | 1^a^ | | 99 | 1.89 | 5.3*10^12^ |  |  |  |  |  | |  |  |
| Evekeo ODT | 1^a^ | | 97 | 0.77 | 2.5*10^8^ |  |  |  |  |  | |  |  |
| Focalin | 2 | | [74, 81] | 0.68 | 43.1 | 2891.1 | 18.8 | 25.7 | 29.2 | 43.0 | |  |  |
| Focalin XR | 2 | | [97, 218] | 0.59 | 562.1 | 1.1*10^7^ | 208.8 | 296.7 | 410.4 | 46.0 | |  |  |
| Intuniv | 2 | | [306, 325] | 0.56 | 81885.0 | 1.5*10^15^ | 24906.6 | 41581.6 | 61326.2 | 69.2 | |  |  |
| Jornay PM | 2 | | [153, 161] | 0.44 | 23.2 | 540.7 | 9.6 | 10.4 | 23.6 | 23.6 | |  |  |
| Kapvay | 2 | | [197, 228] | 0.59 | 55.3 | 5.0*10^8^ | 28.6 | 29.5 | 40.5 | 11.7 | |  |  |
| Metadate CD | 1^a^ | | 314 | 1.04 | 2.3*10^15^ |  |  |  |  |  | |  |  |
| Myadis | 2 | | [257, 263] | 0.81 | 179.1 | 3.6*10^19^ | 83.6 | 121.2 | 99.4 | 110.8 | |  |  |
| QuilliChew ER | 1^a^ | | 85 | 0.76 | 80.8 |  |  |  |  |  | |  |  |
| Quillivant XR | 1^a^ | 132 | | 1.74 | 3.7*10^14^ |  |  |  |  |  |  | |  |
| Ritalin LA | 1^a^ | 39 | | 1.02 | 6.0*10^5^ |  |  |  |  |  |  | |  |
| Strattera | 6 |  | | 0.53 | 32024.2 | 4.3*10 | 11298.2 | 15734.1 | 25161.0 | 7259.8 |  | |  |
| Vyvanse | 2 | [100, 285] | | 1.01 | 2997.2 | 6.4*10^45^ | 1284.8 | 2477.1 | 1421.6 | 89.4 |  | | 675.7 |
| Note: If the heterogeneity Bayes Factor favoured one model over the other, the number is highlighted in bold  ^a^ no meta-analysis performed as this was an individual trial with one arm only | | | | | | | | | | | | | |

## Sensitivity Analysis

Results from the sensitivity analyses are summarized in Table 2. As expected, meta-analytic *BF*s depended to some extent on the scale parameter of the prior, especially for drugs with relatively small effect sizes. *BF*s were smaller when using an optimistic prior ($r=\frac{2}{3}*\sqrt{2}$) compared to when using a more skeptical prior ($r=\frac{1}{6}*\sqrt{2}$). The skeptical prior favours small effect sizes by assigning more weight to effect sizes close to 0. Consequently, evidence for small effect sizes became stronger. Additionally, meta-analytic *BF*s differed depending on the model, i.e., with fixed-effects models resulting in larger meta-analytic *BF*s compared to random-effect models. The random model includes an additional term of variation (i.e., study effect sizes can differ from the true latent population effect), which in turn results in a flatter posterior distribution and lower *BF* for effect sizes further away from 0. Importantly, the qualitative interpretation did not change for different choices of model and/or scale parameter.

Whether or not imputed values were included in the analysis influenced strength of evidence substantially, with meta-analytic *BF*s excluding imputed values suggesting different interpretations (e.g., suggesting strong pro-alternative evidence when the complete analysis suggested ambiguous evidence and vice versa). This is unsurprising as important information is lacking from the analyses excluding imputed values. We frequently imputed values for statistically, non-significant trials (i.e., p >.05) shortly followed by "highly" statistically significant trials (i.e., p < .0001). Excluding these extreme cases is likely to influence the result substantially. Excluding cross-over trials for some drugs approved for the treatment of ADHD did not influence the results qualitatively suggesting that pooling RCTs and cross-over trials did bias the present analysis.

Lastly, we examined how combining results within trials using a fixed-effect model before pooling across trials influenced the results (i.e., “fixed first“). The resulting model-averaged meta-analytic *BF*s were smaller or equal compared to the main analysis. Qualitative interpretation of the result changed for only one drug (i.e., sertraline for PTSD).

# References

Borenstein, M., Hedges, L. V., Higgins, J. P. T., & Rothstein, H. R. (2009). Effect sizes based on means. *Introduction to Meta-Analysis*, 21–32.

Borenstein, M., Hedges, L. V., Higgins, J. P. T., & Rothstein, H. R. (2010). A basic introduction to fixed‐effect and random‐effects models for meta‐analysis. *Research Synthesis Methods*, *1*(2), 97–111.

de Vries, Y. A., de Jonge, P., Van Heuvel, E. D., Turner, E. H., & Roest, A. M. (2016). Influence of baseline severity on antidepressant efficacy for anxiety disorders: Meta-analysis and meta-regression. *British Journal of Psychiatry*, *208*(6), 515–521. https://doi.org/10.1192/bjp.bp.115.173450

De Vries, Y. A., Roest, A. M., De Jonge, P., Cuijpers, P., Munafò, M. R., & Bastiaansen, J. A. (2018). The cumulative effect of reporting and citation biases on the apparent efficacy of treatments: The case of depression. *Psychological Medicine*, *48*(15), 2453–2455. https://doi.org/10.1017/S0033291718001873

Gronau, Q. F., Van Erp, S., Heck, D. W., Cesario, J., Jonas, K. J., & Wagenmakers, E.-J. (2017). A Bayesian model-averaged meta-analysis of the power pose effect with informed and default priors: The case of felt power. *Comprehensive Results in Social Psychology*, *2*(1), 123–138.

Monden, R., de Vos, S., Morey, R., Wagenmakers, E.-J., de Jonge, P., & Roest, A. M. (2016). Toward evidence-based medical statistics: A Bayesian analysis of double-blind placebo-controlled antidepressant trials in the treatment of anxiety disorders. *International Journal of Methods in Psychiatric Research*, *25*(4), 299–308. https://doi.org/10.1002/mpr.1507

Monden, R., Roest, A. M., van Ravenzwaaij, D., Wagenmakers, E.-J., Morey, R., Wardenaar, K. J., & de Jonge, P. (2018). The comparative evidence basis for the efficacy of second-generation antidepressants in the treatment of depression in the US: A Bayesian meta-analysis of Food and Drug Administration reviews. *Journal of Affective Disorders*, *235*, 393–398. https://doi.org/10.1016/j.jad.2018.04.040

Rice, K., Higgins, J. P. T., & Lumley, T. (2018). A re‐evaluation of fixed effect (s) meta‐analysis. *Journal of the Royal Statistical Society: Series A (Statistics in Society)*, *181*(1), 205–227.

Roest, A. M., De Jonge, P., Williams, C. D., De Vries, Y. A., Schoevers, R. A., & Turner, E. H. (2015). Reporting bias in clinical trials investigating the efficacy of second-generation antidepressants in the treatment of anxiety disorders: A report of 2 meta-analyses. *JAMA Psychiatry*, *72*(5), 500–510. https://doi.org/10.1001/jamapsychiatry.2015.15

Scheibehenne, B., Gronau, Q. F., Jamil, T., & Wagenmakers, E.-J. (2017). Fixed or Random? A Resolution Through Model Averaging: Reply to Carlsson, Schimmack, Williams, and Bürkner (2017). *Psychological Science*, *28*(11), 1698–1701. https://doi.org/10.1177/0956797617724426

Turner, E. H., Knoepflmacher, D., & Shapley, L. (2012). Publication Bias in Antipsychotic Trials: An Analysis of Efficacy Comparing the Published Literature to the US Food and Drug Administration Database. *PLoS Medicine*, *9*(3), e1001189–e1001189. https://doi.org/10.1371/journal.pmed.1001189

Turner, E. H., Knoepflmacher, D., & Shapley, L. (2013). Publication bias in antipsychotic trials: An analysis of efficacy comparing the published literature to the us food and drug administration database. *PLoS Medicine*, *9*(3). https://doi.org/10.1371/journal.pmed.1001189

Turner, E. H., Matthews, A. M., Linardatos, E., Tell, R. A., & Rosenthal, R. (2008). Selective publication of antidepressant trials and its influence on apparent efficacy. *New England Journal of Medicine*, *358*(3), 252–260. https://doi.org/10.1056/NEJMsa065779
